# RhoB expression associated with chemotherapy response and prognosis in colorectal cancer

**DOI:** 10.1186/s12935-024-03236-1

**Published:** 2024-02-15

**Authors:** Maria Kopsida, Na Liu, Angeliki Kotti, Jing Wang, Lasse Jensen, Ganesan Jothimani, Camilla Hildesjo, Staffan Haapaniemi, Wen Zhong, Surajit Pathak, Xiao-Feng Sun

**Affiliations:** 1 Department of Oncology and Department of Biomedical and Clinical Sciences, Linköping University, Linköping, Sweden; 2 Department of Gastroenterology, Second Affiliated Hospital of Xi’an Jiaotong University, Xi’an, China; 3 Science for Life Laboratory, Department of Biomedical and Clinical Sciences, Linköping University, Linköping, Sweden; 4 Department of Medical and Health Sciences, Linköping University, Linköping, Sweden; 5 Department of Medical Biotechnology, Faculty of Allied Health Sciences, Chettinad Hospital and Research Institute, Chettinad Academy of Research and Education, Kelambakkam, Tamil Nadu India; 6 Department of Surgery and Department of Biomedical and Clinical Sciences, Linköping University, Norrköping, Sweden

**Keywords:** RhoB, Colorectal cancer, 5-fluorouracil, Oxaliplatin, Survival, Signaling pathway

## Abstract

**Purpose:**

To examine the role of RhoB expression in relation to chemotherapy response, clinical outcomes and associated signaling pathways in colorectal cancer patients.

**Materials and methods:**

The study included 5 colon cancer cell lines, zebrafish embryos and 260 colorectal cancer patients treated with 5-fluorouracil (5-FU) and oxaliplatin (OXL). The methods consisted of CRISPR/Cas9, reactive oxygen species (ROS), caspase-3 activity, autophagy flux, *in-silico* RNA sequencing and immunohistochemistry. Gene expression analysis and pathway analysis were conducted using RNA-seq data.

**Results:**

All cancer lines tested, including SW480, SW480-KO13 (RhoB knockout), SW480-KO55 (RhoB knockout), HCT116 and HCT116-OE (RhoB overexpressed), exhibited cytotoxicity to 5-FU and OXL. RhoB knockout cell lines demonstrated significantly reduced migration compared to the control cell lines. Furthermore, RhoB played a role in caspase-3-dependent apoptosis, regulation of ROS production and autophagic flux. The mRNA sequencing data indicated lower expression levels of oncogenes in RhoB knockout cell lines. The zebrafish model bearing SW480-KO showed a light trend toward tumor regression. RhoB expression by immunohistochemistry in patients was increased from normal mucosa to tumor samples. In patients who received chemotherapy, high RhoB expression was related to worse survival compared to low RhoB expression. Furthermore, the molecular docking analysis revealed that OXL had a higher binding affinity for RhoB than 5-FU, with a binding affinity of -7.8 kcal/mol and HADDOCK predicted molecular interactions between RhoB and caspase 3 protein. Gene-set enrichment analysis supported these findings, showing that enrichment of DNA damage response pathway and p53 signaling in RhoB overexpression treatment group, while the RhoB knockout treatment group exhibited enrichment in the negative regulation pathway of cell migration.

**Conclusion:**

RhoB was negatively associated with chemotherapy response and survival in colorectal cancers. Therefore, RhoB inhibition may enhance chemotherapeutic responses and patient survival.

**Supplementary Information:**

The online version contains supplementary material available at 10.1186/s12935-024-03236-1.

## Background

Colorectal cancer (CRC) is the second leading cause of cancer death for men and women [1]. Surgery is the main option for CRC therapy, and chemotherapy (CT) and/or radiotherapy (RT) could significantly decrease the local relapse and increase survival [2]. However, many tumors resist therapies, and approximately 30% of tumors spread to distant organs, leading to poor prognosis [3]. One of the main reasons is that currently used clinicopathological factors, even tumor stage, cannot precisely provide evidence for clinicians to design and carry out an efficient therapy strategy. Therefore, it is urgent to identify promising biomarkers for a newer approach to increase therapy response.

Ras homolog gene family member B (RhoB) proteins function as a binary switch in a wide range of signal transduction pathways [4]. Rho GTPases are essential signal transducers in signaling pathways that regulate cell proliferation, migration, survival, and death [5]. Rho proteins have various cellular activities and seem to play distinct, sometimes opposing roles in carcinogenesis despite having more than 85% sequence identity [6]. RhoA is essential for contractility and proliferation via multiple growth-related gene expression pathways and actomyosin machinery. In contrast, RhoC plays a vital role in cell–cell adhesion and invasion [7]. RhoB is involved in membrane trafficking and cell survival and has a distinctive endosome localization pattern. It has been proven that members of the Rho family, particularly RhoA and RhoC, have significantly contributed to the development of cancer [8]. However, RhoB attracts growing interest, as its expression is altered in several cancer types. The downregulation of the RhoB in various tumor cell types has led to the hypothesis that it might function as a tumor suppressor [6]. Recent studies have shown that RhoB expression causes apoptosis in cancerous epithelial cells and fibroblasts [9–11]. DNA damage, cytokines, and growth factors might increase RhoB expression and promote oncogenesis [12]. In parallel, other studies demonstrated that RhoB stimulates cell motility and migration or may even promote cancer metastasis [13]. These results indicate the complex and controversial roles of RhoB in cancers. Our earlier research in a clinical trial of preoperative RT in rectal cancer patients showed that RhoB overexpression was associated with later TNM stage, distant recurrence and worse survival in the RT patients but not in non-RT patients. Our study further revealed that RhoB was related to radiation resistance through Akt-FOXM1 [14].

In the current study, we examined the effects of RhoB together with CT on cell lines including knockout and overexpressed cell lines, zebrafish models and in tissue samples from CRC patients. This is the first study to investigate the relationship between RhoB expression, CT response and clinical outcomes in CRC patients as well as to identify the signaling pathway of the RhoB expression with CT response.

## Material and methods

### Cell lines

SW480 colon cancer cell line was obtained from the American Type Culture Collection (ATCC, Manassas, VA). Two SW480 knockout cell lines, SW480-KO16 and SW480-KO55, were generated at our laboratory using the predesigned RhoB-human gene knockout kit via CRISPR (#KN209837, OriGene Technologies, Rockville, MD) [14]. The three cell lines were maintained in Eagles MEM (Sigma-Aldrich, St. Louis, MO) with 10% heat-inactivated fetal bovine serum albumin (GIBCO, Invitrogen, Paisley, UK) and 2 mM L-Glutamine at 37 °C and 5% CO2 at that temperature (Life Technologies, Carlsbad, CA). HCT116 colon cancer cell line was received from the Johns Hopkins University core cell center and kept in McCoy's 5A medium (Sigma-Aldrich) supplemented with 10% heat-inactivated fetal bovine serum albumin (GIBCO, Invitrogen, Paisley, UK) at 37 °C and 5% CO2.

#### Cell transfection

HCT116-OE (RhoB overexpressed) cell line was established as described below. The coding region sequence (CDS) of human *RHOB* was cloned to the *pLVX-ZsGreen-PGK-Puro* vector (Land Liankang Biotechnology Co., Ltd, Guangzhou, China). Cell transfection followed the manufacturer’s instructions (Land Liankang Biotechnology Co., Ltd). Cells of the negative control group were established under the same conditions. The transfection efficiency was measured through Western blot, as shown in Additional file 1: Figure S1.

#### Cell viability assay

Following the manufacturer's instructions, the WST-1 test (Roche, Basel, Switzerland) was used to evaluate the cell viability of the various cell lines after treatment. Briefly, 10,000 cells (SW480, SW480-KO16, SW480-KO55) or 3,000 cells (HCT116, HCT116-OE) were plated in 100 μl complete medium for 24 h before treatment and followed by incubation at 37 °C and 5% CO_2_. Compared to untreated or 0.1% DMSO-treated samples, the proliferation was assessed 72 h after treatment with 5-FU or OXL at increasing doses (vehicle control). Using a microplate reader, the absorbance at 440 nm was determined after adding 10 μl/well WST-1 proliferation reagent (Roche Applied Science) and incubating for 3 h.

#### Boyden chamber migration assay

Following the manufacturer's directions Boyden chamber migration experiments (8 m pore size, Corning, NY) were carried out. In the migration chamber, 1 × 10^5^ cells were seeded, which were then introduced in a well plate containing complete media and incubated for 72 h at 37 °C and 5% CO_2_. After incubation, the cells were then fixed in 4% paraformaldehyde, stained with 0.2% crystal violet in 2% ethanol, and rinsed with phosphate-buffered saline (PBS). A light microscope was used to acquire pictures of moving cells (Zeiss Lab.A1, Jena, Germany). Under an x-100 magnifying lens, the migration rate was evaluated by counting the number of cells that passed through the filter.

#### Detection of reactive oxygen species (ROS)

By using the manufacturer's recommended reactive oxygen species assay kit, intracellular ROS levels were measured (BioVision, Inc., Milpitas, CA). Cells were retrieved and reconstituted in 100 μM dihydro-dichlorofluorescein diacetate (H_2_DCFDA) with serum-free media after a 72-h treatment with 5-FU and OXL. Intracellular H_2_DCFDA was esterified to dichlorodihydrofluorescein, which ROS then oxidized to create the fluorescent substance dichlorofluorescein. The expression level was assessed by measuring fluorescence using plate reader after a 45-min incubation period at 37 °C (Excitation 495 nm, Emission 529 nm).

#### Caspase 3 activity measurement

Caspase 3 activity in SW480, SW480-KO55, SW480-KO16, HCT116 and HCT116-OE cell lines were estimated using a caspase 3 activity assay kit, according to the manufacturer's protocol (BioVision, Inc.). The cells were briefly lysed in a buffer solution containing a caspase 3 sample (BioVision, Inc.). The homogenates of cultured cells were then clarified by differential centrifugation at 10,000 × g, and 4 °C for 10 min and the supernatant was collected. The cell lysates (200 µg) were then introduced to the DEVD substrate conjugate for 2 h at 37 °C followed by supernatant collection. The samples were measured in a microplate reader at an excitation of 405 nm.

#### Autophagy microplate assay

The autophagy flux was determined by using a Cyto-ID autophagy detection kit following the instructions provided by the manufacturer (Enzo, UK). Briefly, 10,000 cells (SW480, SW480-KO16, SW480-KO55) or 3,000 cells (HCT116, HCT116-OE) were seeded and incubated for 24 h in a 96-well plate at 37 °C. Chloroquine (10 μM), Rapamycin (0.5 μM), OXL, and 5-FU (IC_50_ concentrations, Table 1), and negative control were added. The medium was cautiously taken out and discarded after treatment. A dual-color detection solution was applied to each well (100 μL) after cells had been rinsed with 1X Assay Buffer (100 μL). For 30 min at 37 °C, the plate was incubated in the dark. To eliminate excess dye, a new 1X Assay Buffer (100 μL) was added to each well, before which the cells were washed twice with 1X Assay Buffer (200 μL). The Hoechst 33342 Nuclear Stain was assessed with a DAPI filter (Excitation 480 nm, Emission 530 nm), and the CYTO-ID Green detection reagent was measured with a FITC filter (Excitation 340 nm, Emission 480 nm).

**Table 1:** Cell lines treated with 5-flourouracil (5-FU) and oxaliplatin (OXL) at different concentrations (0–60 μM) for 72 h at 37 °C. IC_50_ values correspond to the concentration required to reduce cell growth by 50% compared to control cells. DMSO was used as positive control

Cell lines	IC_50_ values (µM) (Mean ± SEM)	
	5-FU	OXL
SW480	38.5 ± 0.8	21.9 ± 0.4
SW480-KO16	27.2 ± 0.5**	17.8 ± 0.3
SW480-KO55	25.9 ± 0.4**	18.8 ± 0.9
HCT116	51.2 ± 0.3	30.4 ± 0.5
HCT116-OE	54.8 ± 1.1	35.7 ± 0.7

Data presented as mean ± SEM of 3 independent experiments (n = 3)

^*^P < 0.05, **P < 0.01, compared to SW480 and HCT116

#### RNA-seq library generation and sequencing

RNA was extracted using phenol–chloroform method, utilizing kits manufactured by BGI (Shenzhen, China). Nearly 10–50 mg of tissue samples were powdered using liquid nitrogen and transferred into a tube containing 1.5 ml of Trizol reagent. The homogenized samples were centrifuged at 12000xg for 5 min at 4 °C.

The supernatant was transferred to a new tube and mixed with 1.5 ml of Trizol reagent along with 0.3 ml of chloroform/isoamyl alcohol (24:1). For 15 s, the tubes were given a vigorous shake followed by 10 min of centrifugation at 12000 g at 4 °C. The aqueous phase was transferred to a new tube where an equal amount of isopropyl alcohol was added. Further, the tubes were centrifuged at 19,645 g for 20 min at 4 °C. After the removal of the supernatant, 1 ml of 75% ethanol was used to wash the RNA pellet. Later, the air-dried pellet was treated with 25–100 μl of DEPC water depending on the RNA concentration. The RNA library was prepared as per BGI, China’s protocol. Thereafter, Qubit 3.0 Fluorimeter and Agilent 2100 Bioanalyzer were used to quantify the purified libraries. And further sequencing was performed.

#### RNA-seq alignment and quality control

Raw reads containing more than 50% of low-quality bases, defined as bases with a sequence quality of no higher than 5% or adapters read with more than 10% unknown bases, were removed to minimize data noise. After quality control, clean reads were mapped to the reference transcriptome using Bowtie2 [15] with default parameters (performed by BGI, China). The quantification tool for RNA-Seq by Expectation–Maximization was used to compute maximum likelihood abundance estimates for transcripts that were isoforms of the same gene. The fragments per kilobase of exon per million fragments (FPKM) values were calculated according to the standard protocol provided by BGI, China. Differential expression analysis was carried out using a linear method within the Limma R package [16] with Benjamini–Hochberg FDR for multiple hypothesis testing. Genes with a fold change greater than 1 and adjusted p-value below 0.05 were considered statistically significant. A summary of the genome mapping for samples can be found in Additional file 2: Table S1.

#### Gene-set enrichment analysis

We tested the gene ontology (GO) terms and pathways for over-representation in the set of genes that had either significant differential gene expression or at least one significantly differential splicing isoform. We separately tested up- and down-regulated genes. GO was applied to annotate meaningful gene products of biological processes (BP). And Kyoto Encyclopedia of Genes and Genomes (KEGG) were utilized to identify the target genes in biological pathways. We used the cluster Profiler R package [17] to analyze the GO terms and KEGG pathways. Statistical significance was obtained using the Benjamini–Hochberg FDR method (p < 0.05).

### Zebrafish

At the zebrafish facility at Linköping University, transgenic Tg (fli1: EGFP) zebrafish (ZIRC, Eugene, OR) were kept in accordance with standard organizational procedures. Zebrafish embryos were produced by natural mating, and the following morning, post-spawning, the eggs were collected. The eggs were then cleaned, examined for evidence of successful fertilization, and incubated for injection using an E3 medium enriched with 1-phenyl-2-thiourea at 28.5 °C in humidified ambient air.

The cell lines were cultured at 37 °C and 5% CO2 for 24 h after being labeled with 1,1'-dioctadecyl 3,3,3′3'-tetramethylindocarbocyanine (DiI) at a concentration of 5 ng/ml in PBS for 1.5 h. Cells were gathered and introduced into the perivitelline region of 48-h-old embryos after being labeled. The embryos containing tagged cells in the bloodstream were excised after injection. The number of cells injected was the same across all treatment groups and barely varied between embryos. At 28.5 °C, the embryos (20 per group) were incubated in humidified room air. After 24 h, the embryos were given 0.004 percent tricaine anesthesia to observe the cells under a fluorescent microscope (Nikon D-eclipse C1, Tokyo, Japan).

### Patients

The study included 260 patients from the Southeast Health Care Region of Sweden who were diagnosed with CRC between 1984 and 2013, and the detailed information of the patients has been described at the Additional file 2: Table S2.

The RhoB expression was determined by immunohistochemistry in 189 surgical samples of primary tumors, 72 samples of regional lymph node metastases, 143 samples of normal mucosa adjacent to the tumor tissue (histologically free from cancer and taken from the margin of distant surgical resection) and 203 samples of normal mucosa distant to the tumor tissue (histologically free from adjacent between cancer and normal mucosa. The 4 μM tissue microarray slides from paraffin-embedded blocks were deparaffinized in Aqua de Par 10 Ancillary reagent (ADP1002 M, BioCare Medical) for about 20 min. After being kept at 65 °C for 2 h, all sections were then placed into a pressure cooker Decloaking Chamber NexGen configured in a temperature cycle to achieve a maximum of 110 °C for 5 min to perform heat-induced epitope retrieval using Borg Decloaker RTU antigen retrieval solution (BD1000). After cooling, the slides were rinsed in tap water before being washed in tris-buffered saline (TBS). The slides had been washed in TBS before incubating with a rabbit polyclonal primary anti-RhoB antibody (Santa Cruz, catalog no: sc-180) for 30 min at room temperature while being blocked for 5 min by peroxidase. The tertiary antibody MACH4 Universal HRP-Polymer and the secondary antibody MACH4 Universal HRP-Probe were then applied to the slides for 10 min (BRR 4012). TBS was used to wash the slides after cooling down and then rinsed in the gentle flow of running water. The slides were rinsed in TBS and then treated with a peroxidase inhibitor for 5 min to prevent endogenous peroxidase. Following the detection of HRP, the slides were subsequently rinsed with TBS wash buffer. The slides were coated with diaminobenzidine (DAB) chromogen and 1.0 ml of DAB substrate buffer before being incubated for 5 min. The slides were counterstained with Mayer’s hematoxylin staining solution and mounted with PERTEX mounting medium after being rinsed in deionized water.

The immunostaining was evaluated independently by two investigators (MK and CH) without knowledge of clinicopathological data. Staining intensity in or tumor cells was graded according to the following criteria: Immunostaining was evaluated by a semi-quantitative method according to the percentage of positive normal epithelial cells or tumor cells: 0 (negative); 1 (1–25%); 2 (26–50%); and 3 (> 50%). In the case of discrepancy, the sections were re-examined, and a consensus score was reached. Cases with scores of 2 or less were classified statistically as low-expressing groups, whereas those of 3 or more were classified as high-expressing groups.

### Molecular docking analysis of RhoB

#### Molecular interaction of RhoB with chemotherapy drugs

To study the association of RhoB to the chemotherapy response, it is essential to understand the possible molecular interaction between the RhoB protein and the chemotherapeutic drugs through molecular docking analysis. For the molecular docking analysis of the RhoB protein and the chemotherapy drugs, protein, ligand, and grid optimization were prepared using AutoDock Tools 4.2 before initiating the molecular docking analysis [18]. The RhoB protein's x-ray crystallographic 3-dimensional (3-D) structure (PDB id: 2FV8) was retrieved from the protein data bank (PDB) database. The PubChem database was used to obtain the structures of chemotherapy drugs 5-FU (CID: 3385) and oxaliplatin (CID: 9887053). The 3-D structure of 5-FU in SVG format was converted to PDB format during ligand preparation. Since the 3-D structure of OXL is unavailable, the 3-D structure in PDB format was generated using the canonical SMILES. Afterward, the bonds were made rotatable using the torsion tree, optimized, and converted to PDBQT format. The RhoB was then prepared by removing the water molecules, heteroatoms, and co-crystallized solvents. Then, polar hydrogen atoms and Kollman partial charges were added to the structure. The grid box comprising the coordinates x, y, and z, mapping, and size parameter was determined, and molecular docking between the protein and ligands was performed using AutoDock Vina [19]. The results were then analyzed in the Discovery Studio visualization tool [20]. The active binding site amino acid sequences interacting with the chemotherapy drugs were analyzed in the InterPro web server to identify the drug interaction with the binding domain of the protein.

#### Interaction analysis of RhoB with Caspase 3 protein

RhoB and caspase 3 docking was performed on the protein–protein docking web server to identify the molecular interaction in regulating RhoB-mediated caspase 3 expression. Similar to the protein preparation of RhoB, the 3-D structure of caspase 3 (PDB id: 1CP3) was retrieved from the PDB database. Before docking, the water molecules, heteroatoms, protein-bound miRNA, and ligand were removed. The protein–protein docking analysis was performed with RhoB and caspase 3proteins using High Ambiguity Driven Biomolecular Docking (HADDOCK) webserver v2.4 [21, 22]. The HADDOCK will cluster the docked protein structures and the best-docked structure of the topmost cluster based on the lowest HADDOCK score. The molecular interaction between the protein complexes was further analyzed in the PRODIGY webserver [23, 24] and visualized using the PyMOL tool. The 2-D interaction map was generated to identify the interacting amino acids and their type of interaction using LigPlot [25].

### Statistical analysis

All the experiments of cell lines and zebrafish studies were performed with minimum triplicates, if not specified otherwise and presented as mean ± standard deviation or standard error of the mean. Statistical significance was determined using two-way analysis of variance (ANOVA) followed by Tukey's multiple comparison test. *P* < 0.05 were considered statistically significant. Statistical analyses and graphs were made using GraphPad Prism 9. To test differences in RhoB expression levels in primary tumors, adjacent normal mucosa, distant normal mucosa and metastases, the McNemar's test was used. The Chi-squared test was used to compare the clinicopathological factors. The log-rank test was used for the survival analyses, and survival curves were calculated using the Kaplan–Meier method. The outcome for the survival analyses was 10 years OS. Adjusted Hazard ratio (HR) and 95% confidence interval (CI) were calculated using multivariate Cox proportional hazard model. All statistical analyses for the clinical part were performed using Statistica software, with p < 0.05 considered statistically significant.

## Results

### Cell lines

#### Cytotoxicity assay

As shown in Table 1, cytotoxicity evaluation of 5-FU and OXL was performed using WST-1 assay against SW480, SW480-KO16, SW480-KO55, HCT116 and HCT116-OE cells. After 72 h treatment, the two drugs exhibited potent cytotoxicity with IC_50_ values ranging from 17.8 μM to 34.1 μM after treatment with OXL, and 25.9 μM to 54.8 μM after treatment with 5-FU. OXL was more active against all the tested cell lines compared to 5-FU. 5-FU exhibited the lowest IC_50_ value in SW480-KO16 and SW480-KO55 cells compared to SW480, while a similar pattern of toxicity was found after treatment with OXL. IC_50_ value after treatment with 5-FU was 27.2 ± 0.5 in SW480-KO16 cells and 25.9 ± 0.4 in SW480-KO55. After treatment with OXL, the IC_50_ value was 17.8 ± 0.3 and 18.8 ± 0.9 in SW40-KO16 and SW480-KO55, respectively. According to the findings, in both RhoB knockout cell lines, 5-FU and OXL exhibited potent anti-cancer activity, with 5-FU being the best candidate among the two drugs. On the other hand, 5-FU and OXL IC_50_ values were higher in HCT116-OE cells showing a trend compared to HCT116 cells.

These results show that 5-FU and OXL in RhoB overexpressed cells exhibited less cytotoxicity.

#### Migration

**Fig. 1:**
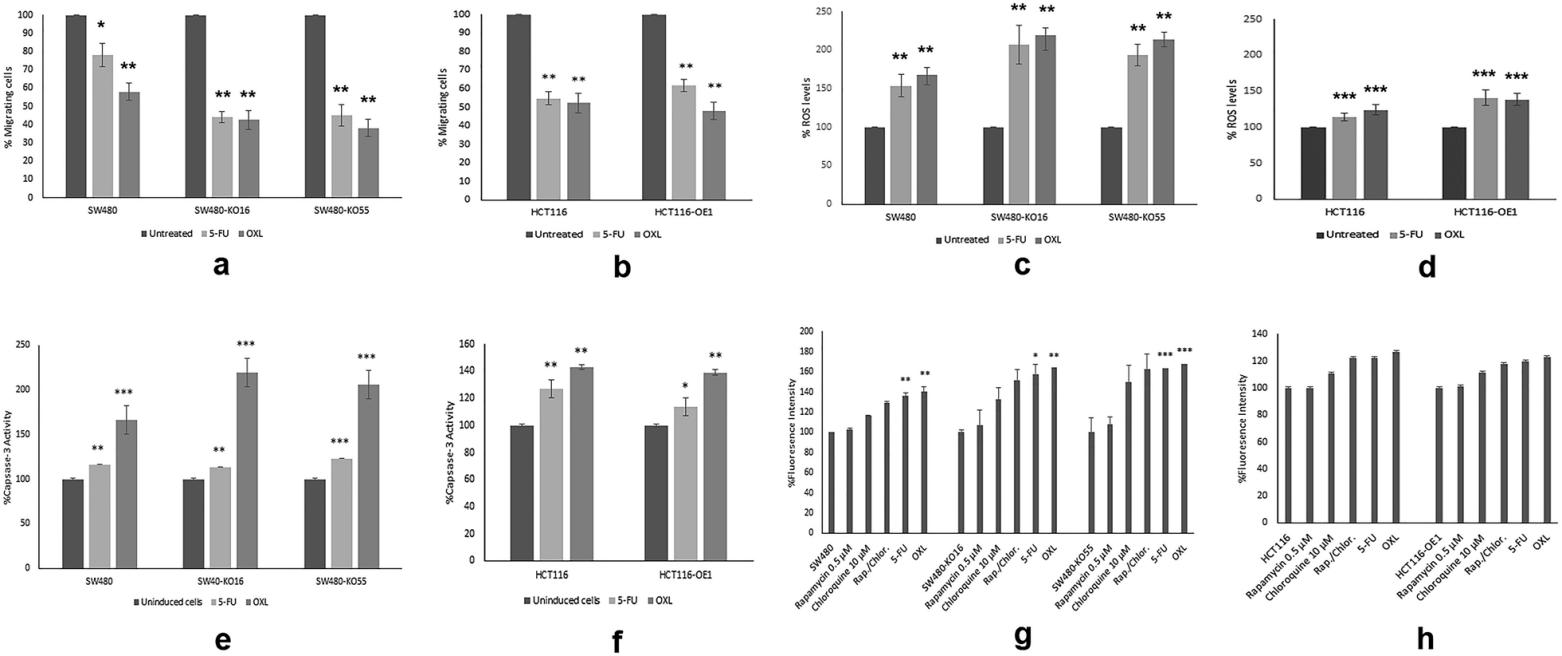
Assessment of 5-fluoouracial (5-FU) and oxaliplatin (OXL) treatment on cell migration, reactive oxygen species (ROS) levels, caspases-3 activity and autophagy in SW480, SW480-KO16, SW480-KO55, HCT116 and HCT116-OE cells. **a**–**b** Cell migration in SW480, SW480-KO16, SW480-KO55, HCT116 and HCT116-OE cells after treatment with 5-FU and OXL (IC_50_ values) for 72 h at 37 °C. Cells were seeded in FBS free medium in migration chambers and were placed in 24 well plates with FBS containing medium. Migration was determined by counting the cells migrated after 72 h. DMSO was used as positive control. Data presented as mean ± SEM of 3 independent experiments (n = 5). *P < 0.05, **P < 0.01, compared to SW480 and HCT116; **c**–**d** Quantification of reactive oxygen species (ROS) levels in SW480, SW480-KO16, SW480-KO55, HCT116 and HCT116-OE cells after treatment with 5-FU and OXL (IC_50_ values) for 72 h at 37 °C. The percentage of ROS production was calculated by measuring the fluorescence intensity. DMSO was used as positive control. Data presented as mean ± SD (n = 3). *P < 0.05, **P < 0.01, ***P < 0.001 compared to SW480 and HCT116, respectively; **e**–**f** Caspase-3 activity in SW480, SW480-KO16, SW480-KO55, HCT116, HCT116-OE1 and HCT116-OE2 cells after treatment with 5-FU and OXL (IC_50_ values) for 72 h at 37 °C. DMSO was used as positive control. Data presented as mean ± SEM (n = 3). *P < 0.05, **P < 0.01, ***P < 0.001, compared to SW480 and HCT116, respectively; and (g-h) Detection of autophagy in SW480, SW480-KO16, SW480-KO55, HCT116 and HCT116-OE cells after treatment with 0.5 µM rapamycin, 10 µM Chloroquine, 0.5 µM Rapamycin + 10 µM Chloroquine, 5-FU and OXL (IC_50_ values). DMSO was used as positive control. Data presented as mean ± SEM (n = 3). *P < 0.05, compared to SW480 and HCT116, respectively

To evaluate whether RhoB was involved in chemotherapy-enhanced migration, we examined the role of RhoB in migration for all the cell lines. By performing the Boyden chamber assay, we found that SW480, SW480-KO16 and SW480-KO55 cells had a significantly lower migration after treatment with 5-FU and OXL than untreated cells (Fig. 1a). Interestingly, the migration rate in HCT116 and HCT116-OE was also significantly decreased after treatment with 5-FU and OXL (Fig. 1b). However, the migration rate did not alter among HCT116 and RhoB overexpressed cell lines after treatment with 5-FU and OXL (Additional file 1: Figure S2b, as within SW480 and RhoB knockout cell lines (Additional file 1: Figure S2a). Matrigel-coated modified Boyden chamber assay was used for examining the invasion status of the above cell lines, but invasive properties were not detected.

The results indicate that RhoB knockout in SW480 cells hinders migration after treatment, RhoB overexpression does not consistently impact migration rates, highlighting the nuanced and context-specific nature of RhoB's role in chemotherapy-induced migration.

#### ROS levels

The production of ROS in all the cell lines was examined after 72 h of treatment with 5-FU and OXL. In SW480, SW480-KO16 and SW480-KO55 cells, ROS levels were significantly increased compared to the untreated cells after treatment with 5-FU and OXL (Fig. 1c). ROS levels in RhoB knockout cells were higher than SW480 cells after treatment with 5-FU and OXL (Additional file 1: Figure S2c).

OXL was found to induce more production of ROS compared to 5-FU among the three tested cell lines. ROS levels in HCT116 and HCT116-OE cells were significantly increased after 72 h of treatment with 5-FU and OXL, but less compared to the ROS production in SW480, SW480-KO16 and SW480-KO55 cells (Fig. 1c, d). The treatments with 5-FU and OXL had similar effects against HCT116 and the RhoB overexpressed cell line after treatment with OXL (Additional file 1: Figure S2d).

These results suggest that RhoB was involved in this stress response, and that 5-FU and OXL induced the production of endogenous ROS within the tested cell lines.

#### Caspase-3 activity

To evaluate whether RhoB was involved in chemotherapy-enhanced cell death, we examined caspase-3-induced activity among all the cell lines examined after 72 h of treatment with 5-FU and OXL. In SW480, SW480-KO16 and SW480-KO55 cells, after treatment with 5-FU and OXL, caspase-3 activity was significantly increased compared to the untreated cells (Fig. 1e). Caspase-3 activity in RhoB knockout cells treated with OXL was higher compared to SW480 cells after treatment with OXL (Additional file 1: Figure S2e). OXL induced caspase-3 activity more compared to 5-FU in these three cell lines (Fig. 1e). Caspase-3 activity in HCT116 and HCT116-OE cells was significantly increased after 72 h of treatment with 5-FU and OXL (Fig. 1f). The treatments with 5-FU and OXL were found to induce more caspase-3 activity within the RhoB overexpressed cell line compared to the HCT116 cell line (Additional file 1: Figure S2f).

These results suggest that RhoB was involved in caspase-3-dependent apoptotic cell death in these cell lines after treatment with 5-FU and OXL.

#### Autophagy flux

To further investigate the role of RhoB in chemotherapy-enhanced cell death, we examined the autophagy flux among all the cell lines after 72 h of treatment with 5-FU and OXL. Compared to the control, autophagy was elevated following 5-FU and OXL treatment in SW480 cell line (Fig. 1g). Autophagy levels were more elevated in both cases compared to the internal controls, Rapamycin and chloroquine or the combination. Autophagy levels were also significantly increased in SW480-KO16 and SW480-KO55 cell lines after treatment with 5-FU and OXL (Fig. 1g), but not compared considerably to SW480 (Additional file 1: Figure S2g). Autophagy was significantly increased in HCT116 and HCT116-OE cell lines after treatment with 5-FU and OXL (Fig. 1h). However, no significant difference in autophagic flux was found within the RhoB overexpressed cell line compared to the HCT116 cell line after treatment with 5-FU and OXL (Additional file 1: Figure S2h).

The results indicate that RhoB positively affected autophagic flux in SW480, SW40KO16 and SW480-KO55 cell lines after treatment with 5-FU and OXL.

#### Gene expression analysis

**Fig. 2:**
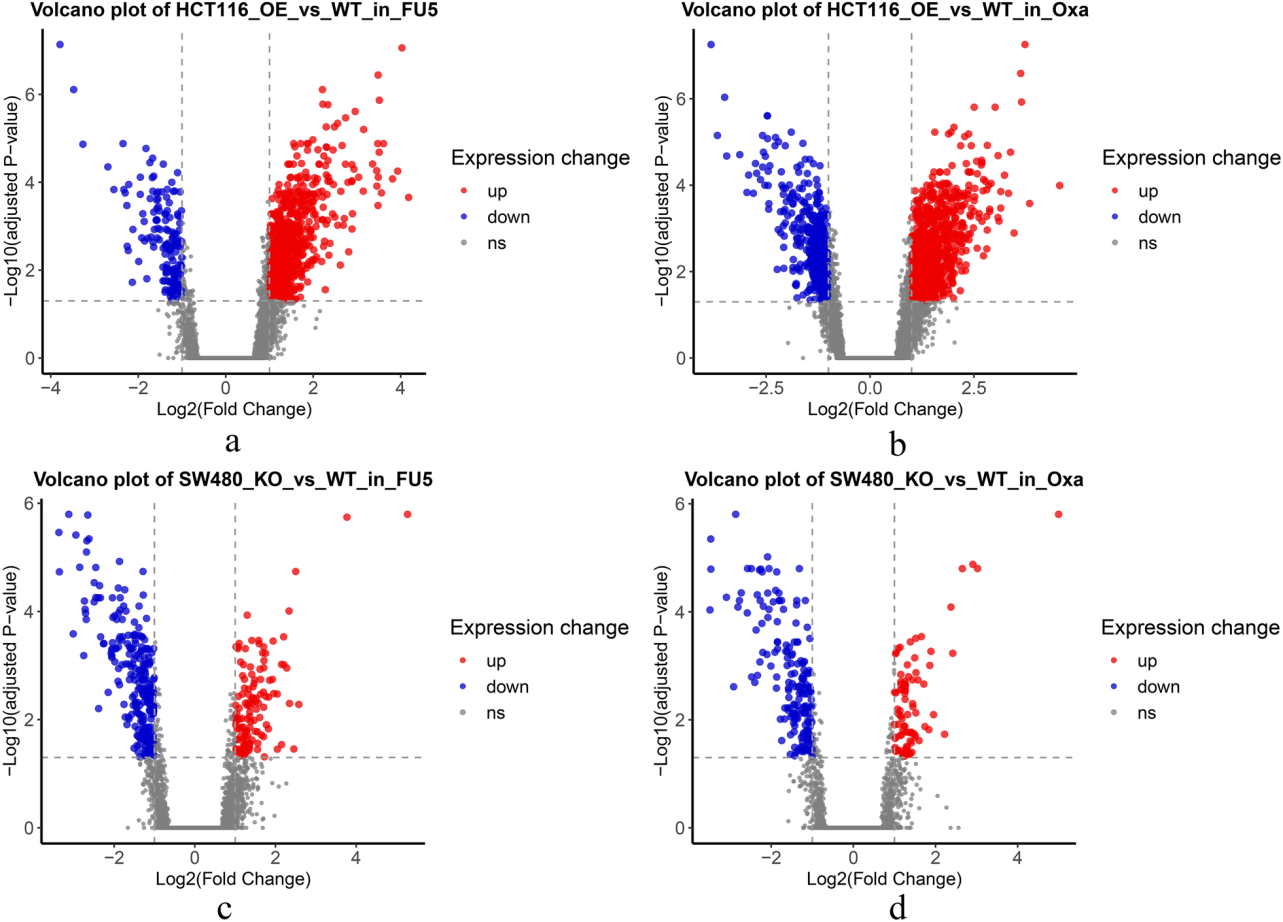
RNA sequencing analysis on the HCT116-OE, HCT116-WT, SW480-KO and SW480-WT cells treated with 5-fluoouracial (5-FU) and oxaliplatin (OXL). Differential gene expression analysis of HCT116 and SW480 cells treated with 5-FU and OXL and represented in the volcano plot. **a** HCT116 OE vs wild type (WT) in 5-FU treatment; **b** HCT116 OE vs WT in oxaliplatin treatment; **c** SW480 knockout (KO) vs WT in 5-FU treatment, and **d** SW480 KO vs WT in OXL treatment

To further examine the role of RhoB at the transcriptomic level, we performed RNA-seq on the HCT116-OE, HCT116-WT, SW480-KO and SW480-WT cells treated with 5-Fu and OXL, respectively. Differentially expressed genes (DEGs) between KO/OE and WT cells were identified using the linear method with an adjusted *P* < 0.05. Volcano plots showing all differentially expressed genes, with genes with log2FC > 1 highlighted. In 5-FU-treated HCT116 cells, 159 down-regulated genes and 713 up-regulated genes were identified in the RhoB over-expressed cell line (HCT116-OE) compared to HCT116-WT (Fig. 2a). In OXL-treated HCT116 cells, HCT116-OE showed 462 down-regulated genes and 896 up-regulated genes compared to HCT116-WT (Fig. 2b). For 5-FU-treated SW480 cells, 253 down-regulated genes and 127 up-regulated genes were identified in RhoB knockout SW480 cells (SW480-KO) compared to SW480-WT (Fig. 2c). In OXL-treated SW480 cells, SW480-KO resulted in the down-regulation of 184 genes and up-regulation of 86 genes compared to SW480-WT (Fig. 2d). The DEGs for each group are listed in Additional file 3: Table S3.

**Fig. 3:**
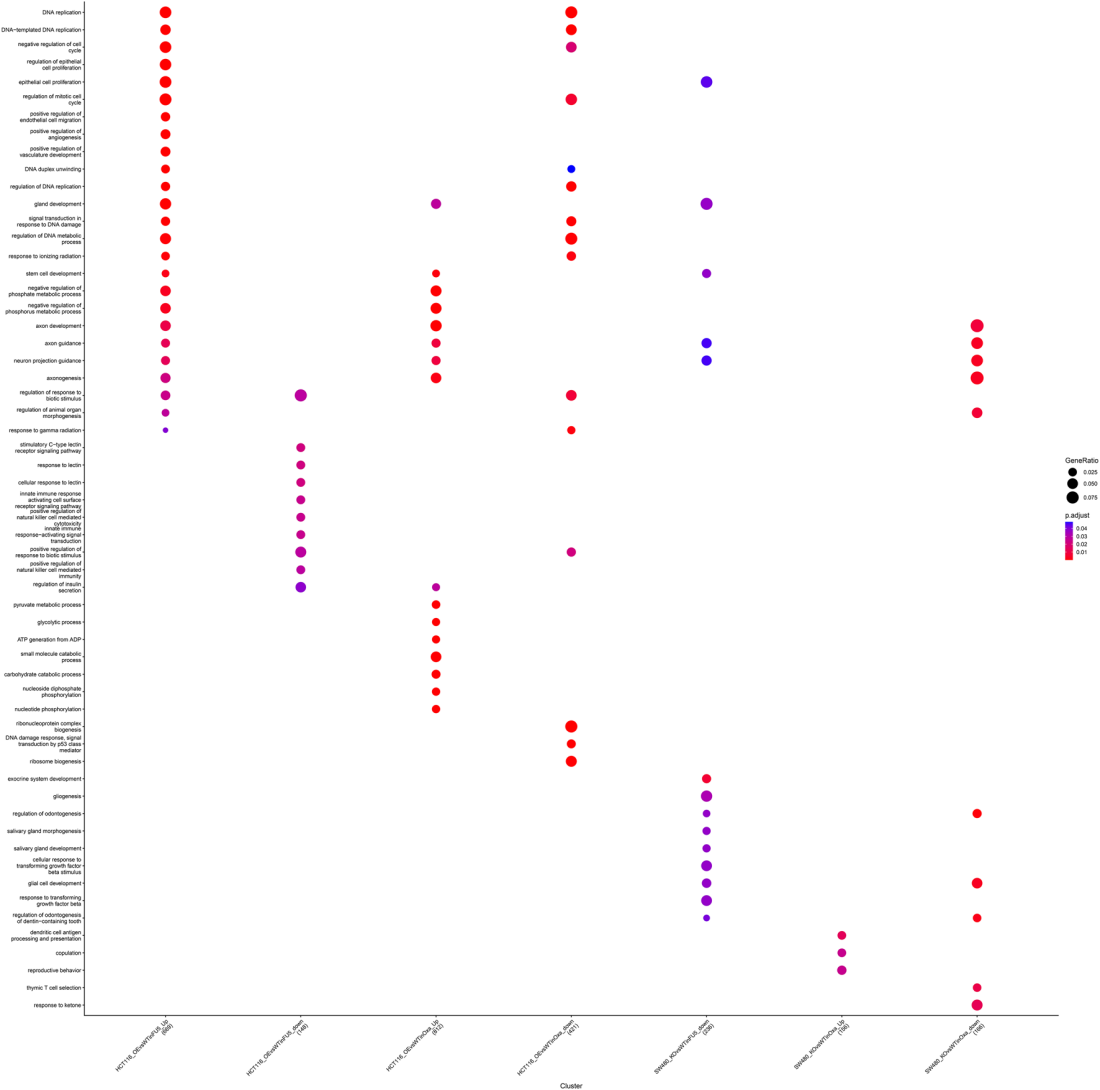
Gene ontology (GO) biological process enrichment analysis of DEGs in HCT116 and SW480 cells after treatment with 5-fluorouracil (5-FU) and oxaliplatin (OXL)

**Fig. 4:**
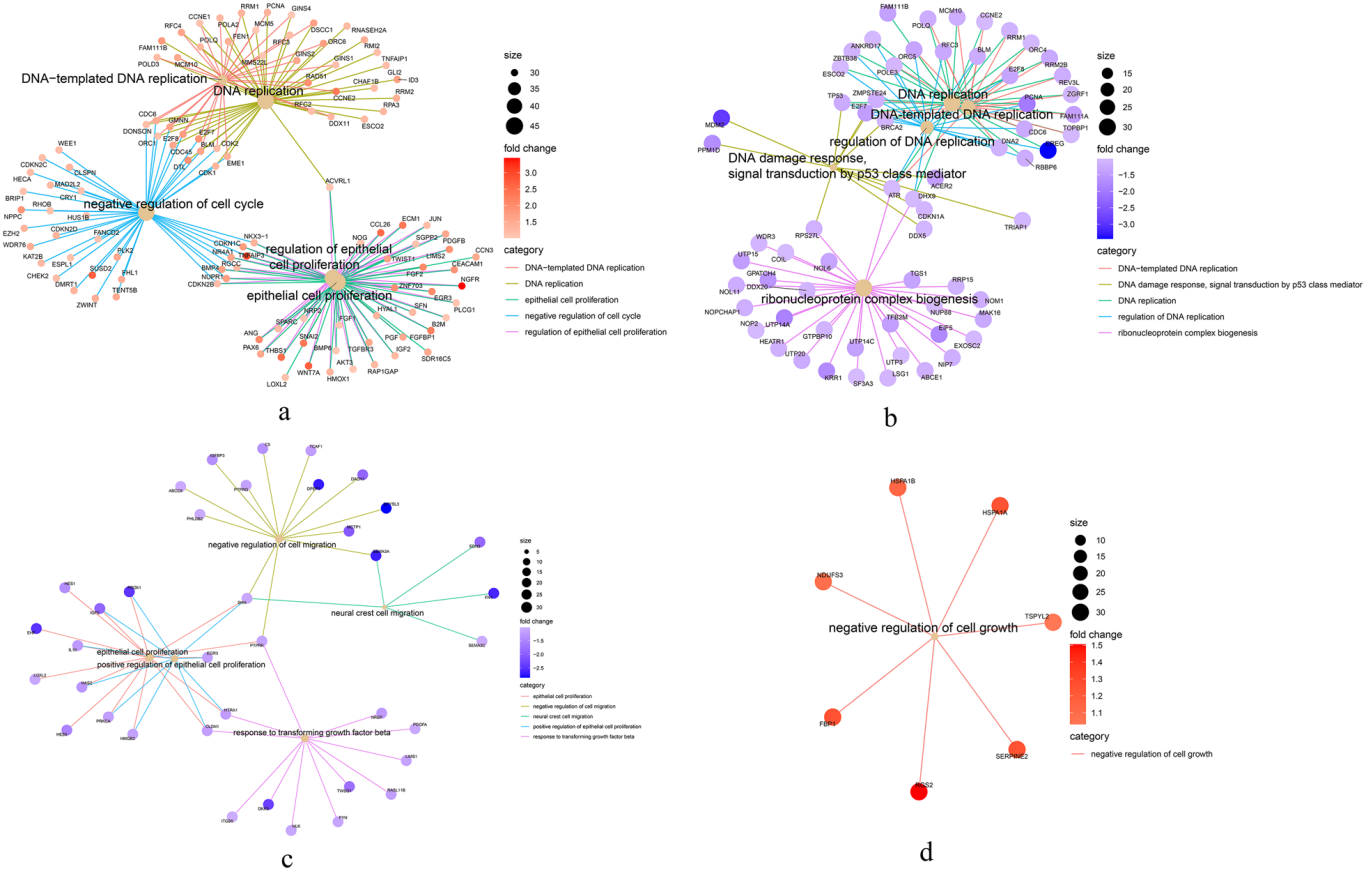
Network analysis of DEGs in HCT116 and SW480 cells treated with 5-fluorouracil (5-FU) and oxaliplatin (OXL). Network analysis of DEGs in HCT116 and SW480 cells treated with 5-fluorouracil (5-FU) and oxaliplatin (OXL). **a** HCT116 OE vs wild type (WT) in 5-FU treatment; **b** HCT116 OE vs WT in oxaliplatin (OXL) treatment; **c** SW480 knockout (KO) vs. WT in 5-FU treatment, and **d** SW480 KO vs WT in OXL treatment

GO and KEGG pathway enrichment analysis based on the DEGs for each of the four groups were conducted (Fig. 3, and Additional file 1: Figure S3). These results indicate that all the DEGs of the four groups are mainly related to the DNA replication and the epithelial cell proliferation processes (Fig. 4a–d), except for the SW480-KOvsWTin5-FU-up group, which had no significant pathway. The KEGG pathway results also revealed that the DEGs of RhoB over-expressed HCT116 cells compared to HCT116-WT are mainly related to the p53 signaling and cell cycle pathways (Additional file 1: Figures S4 and S5).

### Zebrafish

To further investigate the effect of RhoB in CRC, we examined the growth of SW480, SW480-KO55, HCT116 and HCT116-OE cell tumors in vivo using a zebrafish xenograft model. Tumor-bearing zebrafish larvae were incubated for 3 days after injection (day 2 to day 5) and examined under the microscope.

**Fig. 5:**
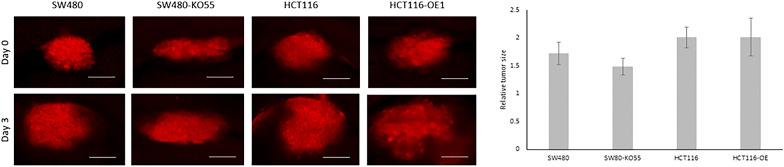
Fluorescent microscopy images and quantification of tumor growth in zebrafish xenograft model. Fluorescent microscopy images of SW480, SW480-KO55, and HCT116 and HCT116-OE tumor xenografts (shown in red). The pictures were taken directly after implantation (day 0) in 2-day old zebrafish *larvae* and 3 days after implantation (day 3). Size-bars in primary tumors correspond to 100 µm. Quantifications of relative tumor volumes indicate the primary tumor size at day 3 relative to day 0

After injecting SW480 and SW480-KO cells into zebrafish larvae, the SW480-bearing zebrafish developed a larger tumor compared to SW480-KO-bearing zebrafish, but the difference in their relative tumor size did not achieve statistical significance. After injecting HCT116 and HCT116-OE cells, both HCT116- and HCT116-OE-bearing zebrafish developed larger tumors compared to SW480- and SW480-KO-bearing (Fig. 5).

The results indicate that SW480-KO-bearing zebrafish displayed a tendency towards tumor regression, although not significant compared to SW480, HCT116 and HCT116-OE cell tumors.

### Patients

#### RhoB expression in distant and adjacent normal mucosa, primary tumor and metastasis

**Fig. 6:**
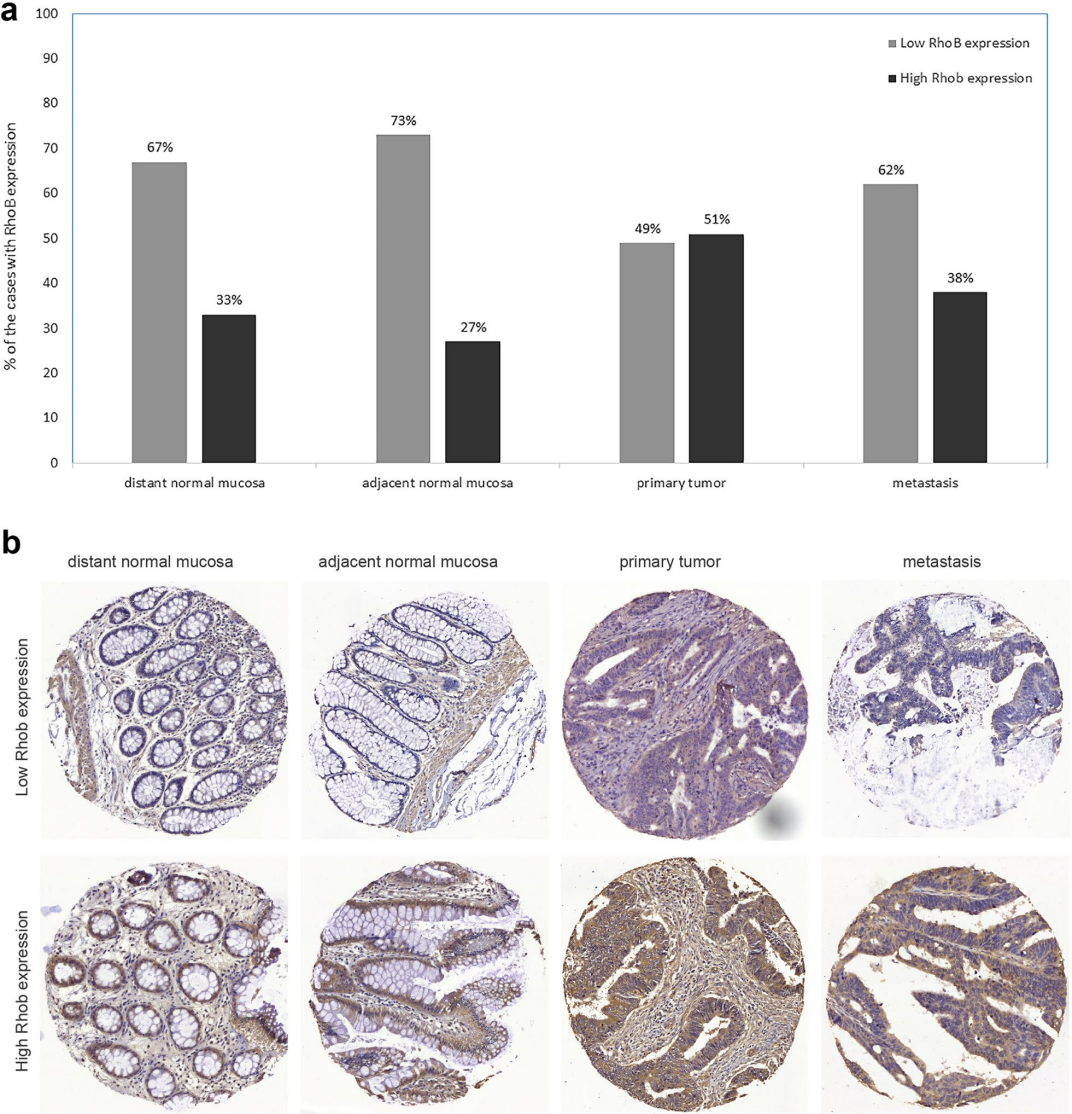
RhoB expression in distant normal mucosa, adjacent normal mucosa, primary tumor, and lymph node metastasis of colorectal cancer patients; **a** the percentage of the cases with RhoB low and high expression and **b** the representative immunohistochemical staining of RhoB low and high expression

Out of 260 patients, 103 samples were taken from distant surgical resection, 143 samples were taken from adjacent normal tissue (both types of the samples were histologically free of cancer), 189 primary cancers and 72 lymph node metastases. All the samples were matched, namely, from the same patients. As shown in Fig. 6a, 135 samples (67%) of distant normal mucosa had low RhoB expression compared to 68 samples (33%) with high RhoB expression; 105 samples (73%) of adjacent normal mucosa showed low RhoB expression compared to 38 samples (27%) with high RhoB expression; 93 primary tumors (49%) had low RhoB expression and 96 (51%) had high RhoB expression; 45 metastases (62%) had low RhoB expression compared to 27 cases (38%) with high RhoB. RhoB expression was increased from distant normal mucosa to primary tumor (McNemar p < 0.001) and from adjacent normal mucosa to tumor (McNemar p < 0.001). There was no difference in RhoB expression between the primary tumor and metastasis (McNemar, p = 0.502). The Fig. 6b shows the RhoB expression of distant normal mucosa, adjacent normal mucosa, primary tumor and metastasis in the lymph node.

The results show that RhoB expression was increased in tumors compared to normal mucosa samples.

#### RhoB expression in primary tumors and survival

**Fig. 7:**
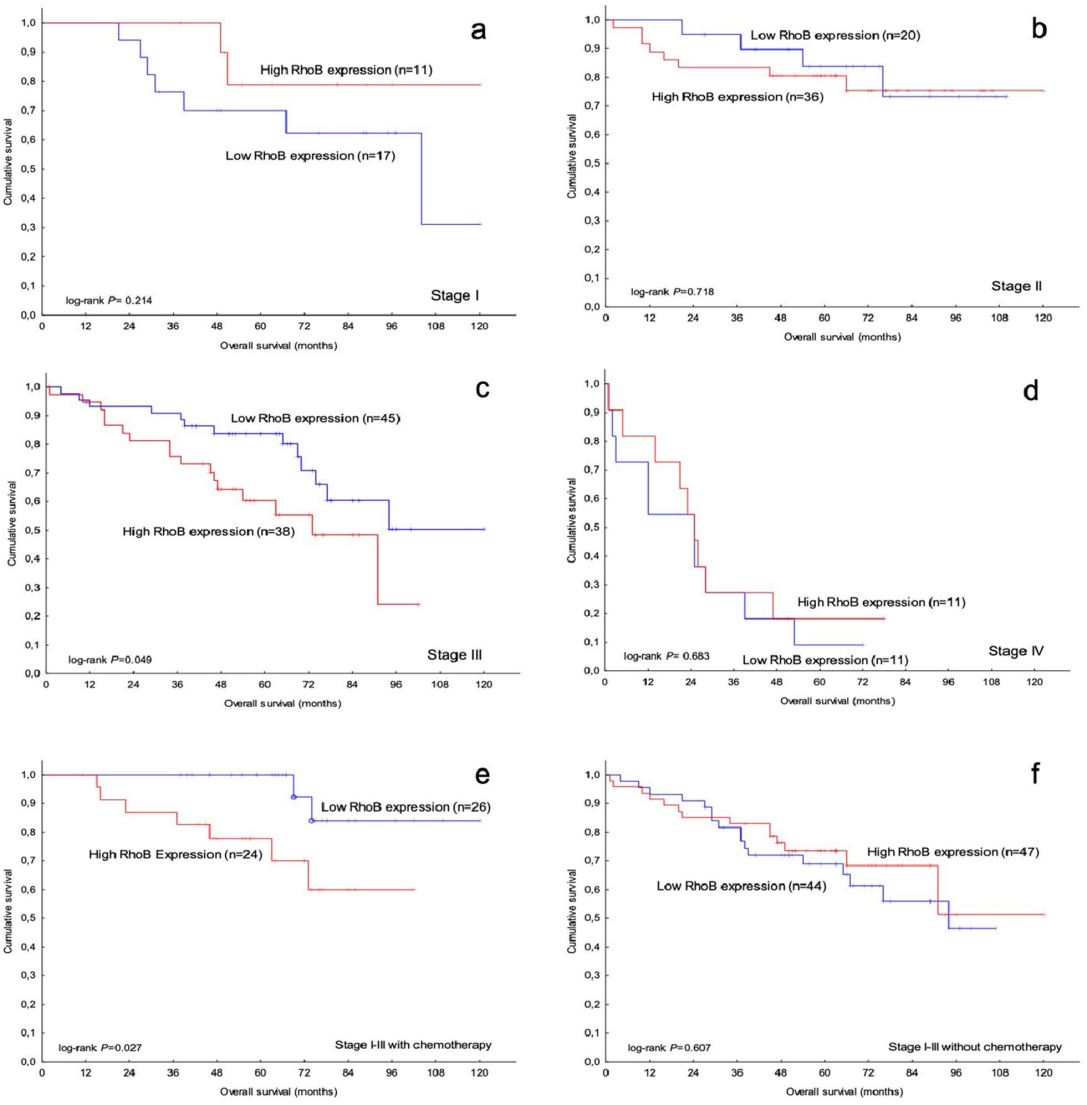
RhoB expression in colorectal cancer in relation to patient overall survival with different stage and treatment; **a** stage I; **b** stage II; **c** stage III and **d** stage IV; as well as **e** stage I-III with chemotherapy and **f** stage I-III without chemotherapy

Next, we investigated the association of RhoB expression in primary tumors with overall survival (OS). High RhoB expression was significantly related to worse OS compared to low RhoB expression in patients with stage-III disease (n = 83, log-rank p = 0.049, Fig. 7c), and there was no such association in stage I (n = 28), II (n = 56) or IV (n = 22), (log-rank p > 0.05; Fig. 7a, b, and d). The difference in OS in stage III remained significant after adjusting for age and location of the primary tumor (HR, 2.70; 95% CI 1.19–6.11, p = 0.018).

We further examined the association between RhoB expression in primary tumors and OS in patients with stage I-III disease who received or did not receive preoperative and/or adjuvant chemotherapy, respectively. Among patients who received chemotherapy, those with high RhoB expression had worse OS than those with low RhoB expression (log-rank P = 0.027; Fig. 7e). No such association was present among patients who did not receive chemotherapy (log-rank P > 0.05; Fig. 7f).

The results show that increased RhoB expression was related to worse survival compared to low RhoB expression in patients who received chemotherapy.

### Molecular docking analysis of RhoB and chemotherapy

#### Molecular interaction of RhoB with chemotherapy drugs

**Fig. 8:**
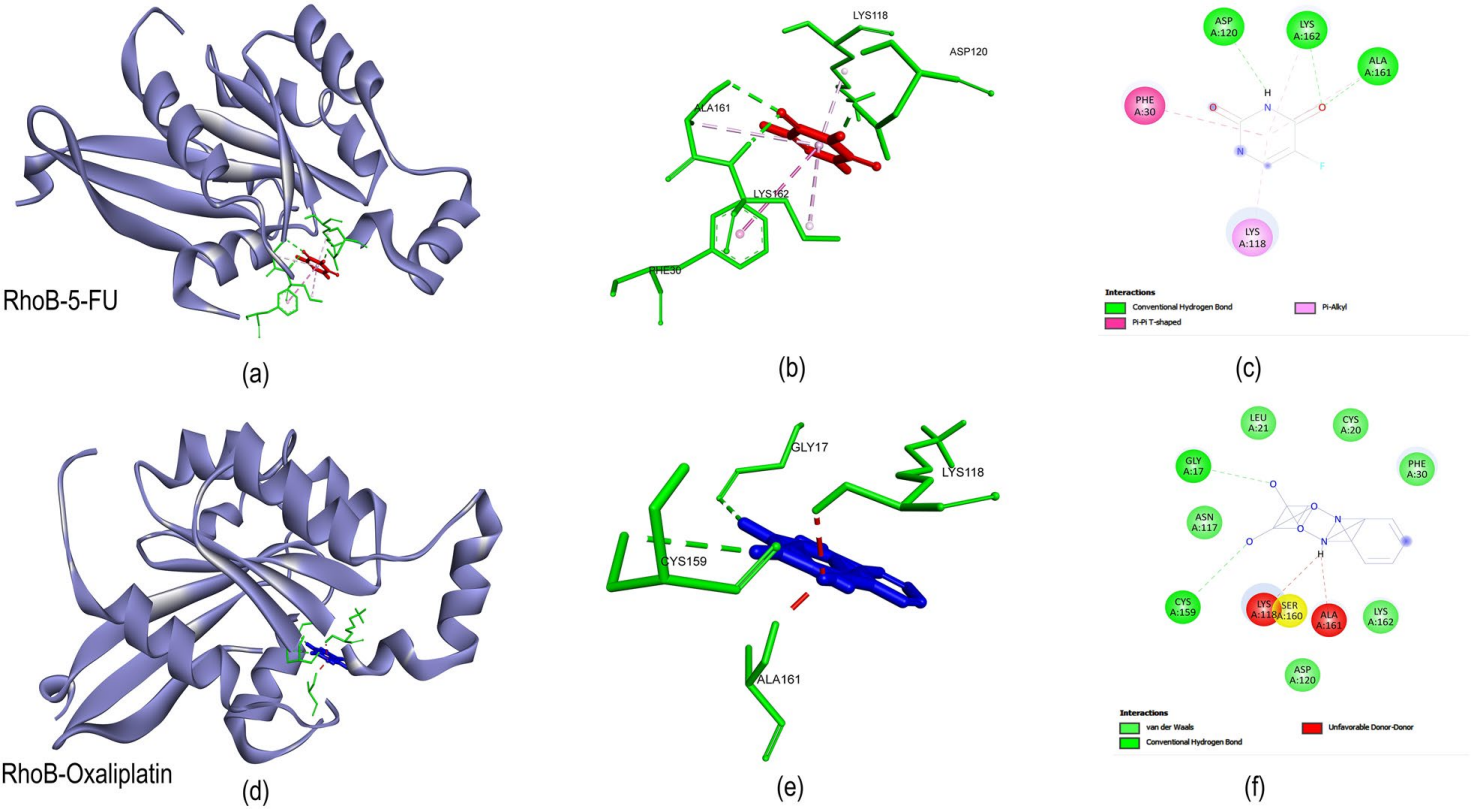
Molecular Docking study **a**–**c** molecular interaction analysis of RhoB protein with 5-fluorouraci (5-FU) by molecular docking; **d**–**f** molecular interaction analysis of RhoB protein with oxaliplatin (OXL) by molecular docking

Given the findings above indicating the role of RhoB in developing chemoresistance in patients and preventing apoptosis in cells overexpressing RhoB treated with chemotherapy drugs, it is necessary to investigate the molecular interaction of 5-FU and OXL to understand its role better. The molecular docking analysis revealed that the OXL had a better binding affinity with the RhoB protein with a least binding energy of −7.8 kcal/mol (Additional file 2: Table S4). The OXL had 2 hydrogen bonds with RhoB amino acid sequences of GLY 17 CYS 159 and 6 Van der Waals interactions with the amino acids LEU 21, CYS 20, PHE 30, ASN 117, ASP 120, and LYS 161. At the same time, the 5-FU had a lesser binding affinity with RhoB having a binding energy of -5.2 kcal/mol. The 5-FU had 3 hydrogen bonds with ASP 120, ALA 161, and Lys 162 amino acids, and 2 hydrophobic interactions with PHE 30 and LYS118 amino acids of RhoB protein. The molecular interaction of RhoB with 5-FU and OXL is depicted in Fig. 8. The InterPro analysis revealed that amino acid sequences from 5 to 159 belong to the small GTP binding domain.

These results indicate that OXL has an advantage of a better interaction with RhoB with higher binding affinity compared to 5-FU.

#### Molecular interaction of RhoB with caspase 3 protein

In response to the increased caspase 3 activity in RhoB knockout cells and in RhoB overexpressed cells treated with OXL, the molecular interaction between the RhoB protein and caspase 3 was performed. The HADDOCK results displayed a significantly higher binding affinity between the RhoB and caspase 3 proteins with a HADDOCK score of −105.1 ± 27 and a z-score 0f −1.7. HADDOCK clustered 162 structures in 8 cluster (s), which represents 81% of the water-refined models HADDOCK generated. The topmost cluster having the least HADDOCK scores is considered the most reliable structure in determining molecular interactions. More importantly, Van der Waals energy and electrostatic energy play a significant role in determining the structural stability of the interacting proteins. The RhoB and caspase 3 protein complexes had highly electrodynamic stability with electrostatic energy of 34.2 ± 34.2 kcal/mol and Van der Waals energy of −56 ± 5.2 kcal/mol, shown in Additional file 2: Table S5. The complete haddock score of all clusters generated was mentioned in Additional file 2: Table S6 and plotted in Additional file 1: Figure S6a-f.

**Fig. 9:**
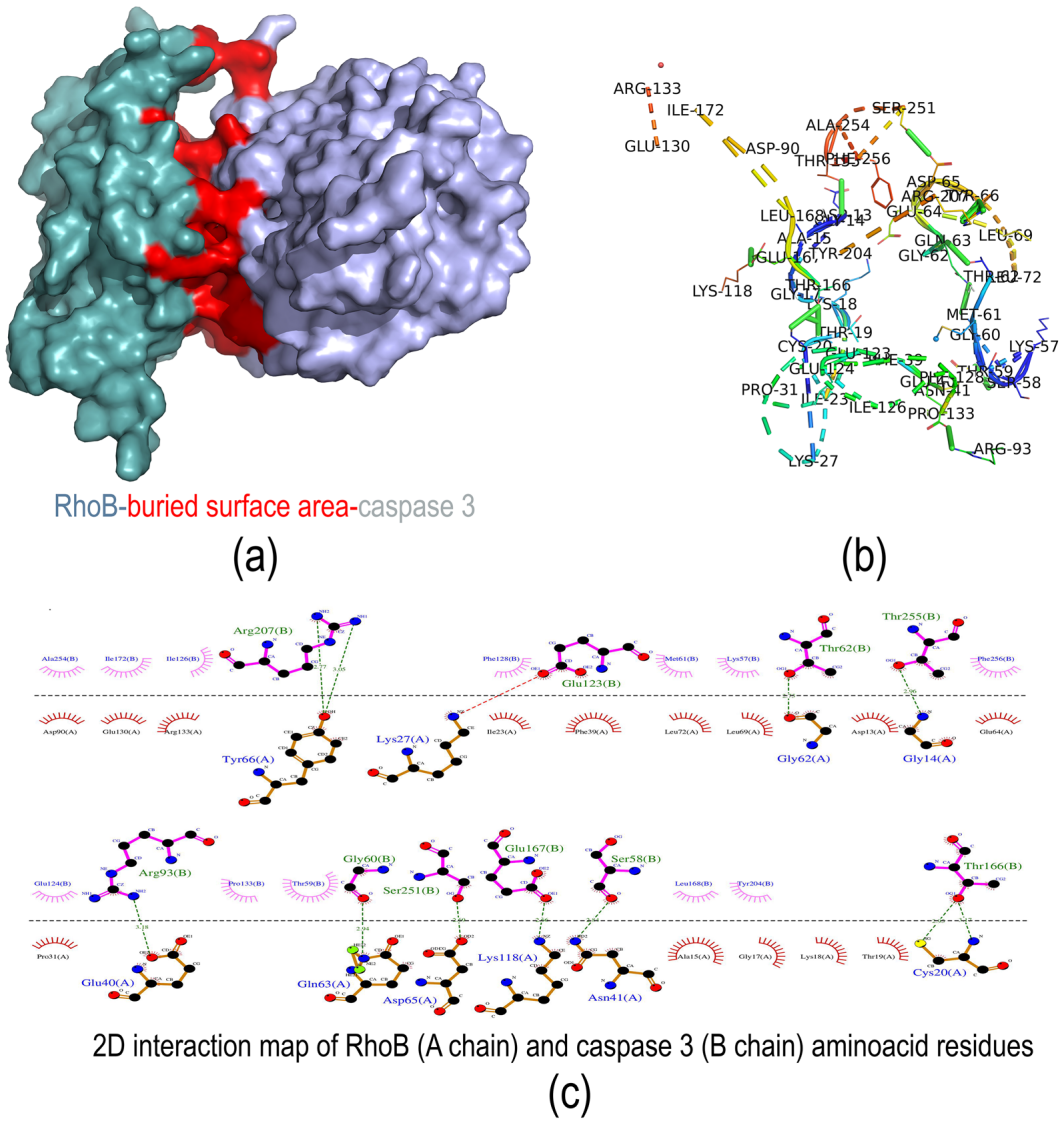
Protein interaction study **a**–**b** protein–protein interaction analysis of RhoB protein with caspase 3 protein using HADDOCK and visualized with PYMOL; and **c** 2D interaction map of interacting amino acids of RhoB protein with caspase 3 protein using LigPlot

The overall binding affinity of RhoB with caspase 3 was determined by ProDIGY analysis. The results showed that the RhoB and caspase 3 complexes had highly stable binding energy with the ΔG value of −12.2 kcal/mol and the dissociation constant, KD (M) value of 1.1 × 10^–9^, mentioned in Additional file 2: Table S6. It also revealed that 96 amino acid residues of each protein were actively involved in the molecular interaction (Additional file 2: Table S7). Among the amino acids interacting with each other, 9 amino acids had complete electrostatic interaction (charged-changed), 11 amino acids had charged-polar, 29 amino acids had charged-apolar interaction, and 7 amino acids had polar-polar interaction, 24 amino acids had apolar-polar, and 16 amino acids had apolar-apolar interaction. The overall interaction of RhoB and caspase 3 with the buried surface area is depicted in Fig. 9a. The amino acid residues involved in the protein–protein interaction (Additional file 2: Table S7) are shown in Fig. 9b. Figure 9c illustrates the key amino acids involved in the hydrogen bonding and hydrophobic interactions between the macromolecules.

## Discussion

The present study has demonstrated that high RhoB expression in patients with stage III tumors that received chemotherapy had worse survival compared to lower RhoB expression; there was no such association in patients who did not receive chemotherapy. The evidence was further validated in zebrafish and cell models. We observe that RhoB knockout cell lines exhibited increased sensitivity to the drug treatments concerning cellular proliferation and apoptosis. Furthermore, gene set enrichment analysis revealed that the differentially expressed genes in the RhoB-treated group were predominantly enriched in the signaling pathways related to cellular proliferation, migration, and p53, compared to the control group.

Our previous study revealed an independent association between RhoB overexpression and poor survival in rectal cancer patients who underwent radiotherapy. In RhoB knockout cells, the expression of FOXM1 was reduced. Downregulation of FOXM1 occurred in RhoB knockout cells, contributing to reduced survival rates and diminished migration and invasion capabilities following radiation. Among patients undergoing radiation therapy, RhoB overexpression correlated with elevated FOXM1 levels, advanced tumor, local and distant metastasis, and independently predicted poor survival, irrespective of other clinical factors [14]. Interestingly, the current findings demonstrate that RhoB overexpression was also associated with worse survival in patients who received chemotherapy. Conclusively, the expression of RhoB significantly impacts the response of CRC patients to either radiotherapy or chemotherapy, suggesting that RhoB provides new insights into improving therapeutic interventions for CRC.

The molecular docking results suggested that the OXL had a better affinity with a maximum of 8 amino acid residues interacting with the ligand. Though OXL had better binding energy, 5-FU also had a negative binding energy with 3 hydrogen bonds and 2 hydrophobic interactions with RhoB. The molecular interaction of OXL with RhoB with the least binding energy added weightage to the strong association between the RhoB protein and the chemotherapeutic drug. The binding of OXL to the pocket region of RhoB had maximum interactions with the amino acid residues compared to the 5-FU suggesting that the OXL might be the key interactor in regulating the RhoB expression and its function. In coherence, the docking analysis of the RhoB protein with 5-FU and OXL showed that modulating the expression of RhoB with OXL might inhibit the molecular interaction between the RhoB and caspase 3 protein to induce apoptosis in tumor cells. Moreover, most of the interacting amino acids of RhoB with 5-FU and OXL were classified in the small GTP binding domain, indicating that the chemotherapy drugs might inhibit the function of RhoB regulating cytoskeletal reorganization, cell polarity, cell cycle progression, and gene expression in a cell. In elucidating the regulation of apoptotic cascade with the 5-FU and OXL through the RhoB-mediated response, it is essential to delineate the molecular interaction between the RhoB and caspase 3 proteins. RhoB-caspase 3 complexes showed a higher structural and electrodynamic stability indicating that the RhoB might involve in the negative regulation of apoptosis. The increase in the caspase 3 activity on RhoB knockout cells provided additional weightage. HADDOCK results predicted the molecular interactions between the RhoB and capsase3 proteins. The hydrogen bonds between the RhoB and caspase3 amino acid residues indicate a stronger binding affinity. In addition, the maximum number of hydrophobic interactions provides better stability and dynamics to the protein complexes. Further, the HADDOCK analysis in the present study has shown that RhoB had a strong association with the mitochondrial-mediated apoptotic regulatory caspase 3 proteins.

The influence of drug-induced ROS on apoptotic cell death has been utilized extensively in developing effective cancer chemotherapy strategies. Numerous studies examining the effect of anticancer agents on CRCs have already demonstrated that most of these compounds influence ROS [26]. According to our findings, 5-FU and OXL probably caused ROS production in the cells, and RhoB may be involved in the stress response process. However, this pathway is yet to be fully understood; this might be the primary mode of action for many drugs used in anti-cancer therapy. It is still unclear how cellular apoptosis affects the clinical outcome in CRCs. To delve deeper into the involvement of RhoB in chemotherapy-induced cell death, we examined the autophagy flux in cells following treatment with 5-FU and OXL. This evaluation validated the correlation between RhoB and caspase 3 activity in cells subjected to chemotherapeutic drug treatment. The outcomes showed that RhoB positively impacts autophagic flux in the SW480, SW40KO16, and SW480-KO55 cell lines following 5-FU and OXL treatment. Moreover, the SW480-bearing zebrafish tended to develop a larger tumor than the SW480-KO-bearing zebrafish after being injected with SW480 and SW480-KO cells. As a result, the findings showed that RhoB was involved in caspase-3-dependent apoptosis in the cells after treatment with 5-FU and OXL.

To further investigate the involvement of RhoB at the transcriptomic level, we conducted RNA-seq analysis on HCT116-OE, HCT116-WT, SW480-KO and SW480-WT cells treated with 5-Fu and OXL, respectively. We observed numerous upregulated and downregulated gene expression levels across the groups (Additional file 3: Table S3). We further carried out GO and KEGG pathway enrichment analysis for the groups based on the DEGs. Our results demonstrated that SW480 vs. SW480-KO16 and HCT116 vs. HCT116-OE1, had comparable biological functions, cellular components, and different signaling pathways. Most genes essential for cellular processes were down-regulated in the SW480 vs. SW480-KO16 group, whereas most genes are up-regulated in the HCT116 vs. HCT116-OE1 group. In coherence, our findings suggest that RhoB was crucial for all tested cell lines to migrate after 5-FU and OXL treatment. In addition, the MAPK signaling pathway and cell adhesion molecules were the most representative KEGG pathways associated with cancer for the SW480 group in comparison to SW480-KO16. Whereas most genes were up-regulated in the HCT116 vs. HCT116-OE1 group. In coherence, our findings suggest that RhoB is crucial for all tested cell lines to migrate after 5-FU and OXL treatment. However, signaling pathways like MAPK, TNF, Ras, and PI3K-Akt emerged as the most prominent KEGG pathways associated with the comparison between HCT116 and HCT116-OE1. Thus, caspase-3 activity results showed that RhoB was involved in caspase-3-dependent apoptotic cell death and positively influenced autophagic flux in 5-FU and OXL-treated SW480, SW40KO16, and SW480-KO55 cell lines. Further, we counted the RhoB OE up-regulated genes and KO down-regulated genes in the 5-FU-treated group and OXL treated group, respectively. In the 5-FU treated group, there were 20 OE up-regulated genes that overlapped with KO down-regulated genes. Similarly, 17 OE up-regulated genes overlapped with KO down-regulated genes in OXL treated group (Additional file 4: Table S8). We merged these two overlapping gene sets (Total 31 genes) to perform GO biological process and KEGG enrichment analysis. Many pathways related to cell proliferation and growth as well as hair development were found (Additional file 5: Table S9, Additional file 1: Figure S7). This is consistent with the reported role of RhoB in regulating cell proliferation, growth, and transformation [27]. Adly et al*.* [28] found that RhoB protein was strongly expressed in the various elements of the human scalp skin and hair follicles [28]. That was also consistent with our results.

Our comprehensive analysis reveals that RhoB plays a pivotal role in modulating the response of CRC cells to chemotherapy. While RhoB overexpression in HCT116-OE cells demonstrated resistance to 5-FU and OXL, the intricate relationship between RhoB, ROS production, and caspase-3 activity underscores the complexity of its involvement in chemotherapy response. Moreover, to assess the impact of RhoB on the cellular response, RhoB knockout cell lines (RhoB KO 16 and RhoB KO 55) were generated from SW480 cells through the implementation of the CRISPR/Cas9 system and the protein expression of RhoB was shown in Additional file 1: Figure S1. The successful establishment of these knockout cell lines was confirmed by the complete depletion of RhoB expression. Notably, this genetic manipulation did not induce significant alterations in the expression levels of closely related GTPases, RhoA and RhoC, underscoring the specificity of RhoB knockout in this experimental model [14].

Reviewing the results of previous studies on the role of RhoB in cancers, RhoB has been implicated in both oncogenic and tumor suppressor functions, highlighting its complex involvement in cancer development and progression. In some studies, RhoB exhibits oncogenic properties by promoting cell proliferation and growth, and reducing therapy response, resulting in poor clinical outcomes including primary tumor metastasis and short survival of patients including colorectal cancers [14, 29, 30]. On the other hand, RhoB has demonstrated tumor suppressor characteristics by inhibiting cell invasion and metastasis, and enhancing therapy response, leading to better clinical outcomes such as bladder, ovarian and head-neck cancers [31–34]. These controversial results may arise from variations in tumor types, animal and cell models, along with the diverse treatments and techniques employed in the different studies. Besides, comprehending the dualistic nature of RhoB in cancers necessitates elucidating the biological factors that govern its behavior through different signaling pathways not only in tumor cells but also tumor cell microenvironment.

## Conclusion

In conclusion, the results of this study demonstrate an upregulation of RhoB expression in tumors, and importantly, an independent association between increased RhoB expression and unfavorable survival among stage III patients who underwent chemotherapy. These findings suggest that targeting the RhoB pathway could provide a promising approach for precise chemotherapy in CRC patients.

### Supplementary Information

**Additional file 1: Figure S1.** Figure showing the protein level of RhoB in all the selected cell lines. **Figure S2.** Figure showing the statistical differences of RhoB WT *vs* KO/OE cell lines. **Figure S3.** KEGG pathway analysis of DEGs in HCT116 and SW480 cells after treatment with 5-fluorouracil (5-FU) and oxaliplatin (OXL). **Figure S4.** KEGG pathway network analysis of DEGs in HCT116 cells after treatment with 5-fluorouracil (5-FU). **Figure S5.** KEGG pathway network analysis of DEGs in HCT116 cells after treatment with oxaliplatin (OXL). **Figure S6.** The overall haddock score of all clusters generated for the RhoB and caspase 3 interaction. **Figure S7.** Gene ontology (GO) biological process and KEGG pathway analysis of overlapping RhoB OE up-regulated and KO down-regulated genes.

**Additional file 2: Table S1.** Genome mapping summary of samples. **Table S2.** Characteristics of colorectal cancer patients. **Table S4.** Molecular docking analysis of RhoB and chemotherapy drugs with the interacting amino acid residues. **Table S5.** Molecular docking analysis between RhoB and caspase 3 proteins. **Table S6.** Protein–protein docking results of complete clusters generated from HADDOCK. **Table S7.** Interacting amino acid residues of RhoB and oxaliplatin.

**Additional file 3: Table S3.** Complete list of differentially expressed genes in the four comparison groups.

**Additional file 4: Table S8.** The list of overlapping genes between RhoB OE up-regulated and KO down-regulated genes.

**Additional file 5: Table S9.** Results of the gene ontology (GO) biological process and KEGG enrichment analysis.

## Data Availability

The data generated in the present study may be requested from the corresponding author.

## Abbreviations

3-D: 3-Dimensional
5- FU: 5-Fluorouracil
ATCC: American type culture collection
BP: Biological processes
CI: Confidence interval
CRC: Colorectal cancer
CT: Chemotherapy
DAB: Diaminobenzidine
DEGs: Differentially expressed genes
DiI: Dioctadecyl 3, 3,3′3’-tetramethylindocarbocyanine
GO: Gene ontology
H_2_DCFDA: Dihydro-dichlorofluorescein diacetate
HADDOCK: High ambiguity driven biomolecular docking
HR: Hazard ratio
KEGG: Kyoto encyclopedia of genes and genomes
OXL: Oxaliplatin
PBS: Phosphate-buffered saline
RhoB: Ras homolog gene family member B
ROS: Reactive oxygen species
RT: Radiotherapy
TBS: Tris-buffered saline
