# Supplementary material for: RhoB expression associated with chemotherapy response and prognosis in colorectal cancer
Source: Cancer Cell Int. 2024 Feb 15;24:75. doi: 10.1186/s12935-024-03236-1 (PMC10867990; doi:10.1186/s12935-024-03236-1)
Supplement: Supplementary file 1 — Additional file 1: Figure S1. Figure showing the protein level of RhoB in all the selected cell lines. Figure S2. Figure showing the statistical differences of RhoB WT vs KO/OE cell lines. Figure S3. KEGG pathway analysis of DEGs in HCT116 and SW480 cells after treatment with 5-fluorouracil (5-FU) and oxaliplatin (OXL). Figure S4. KEGG pathway network analysis of DEGs in HCT116 cells after treatment with 5-fluorouracil (5-FU). Figure S5. KEGG pathway network analysis of DEGs in HCT116 cells after treatment with oxaliplatin (OXL). Figure S6. The overall haddock score of all clusters generated for the RhoB and caspase 3 interaction. Figure S7. Gene ontology (GO) biological process and KEGG pathway analysis of overlapping RhoB OE up-regulated and KO down-regulated genes. [file 12935_2024_3236_MOESM1_ESM.docx]

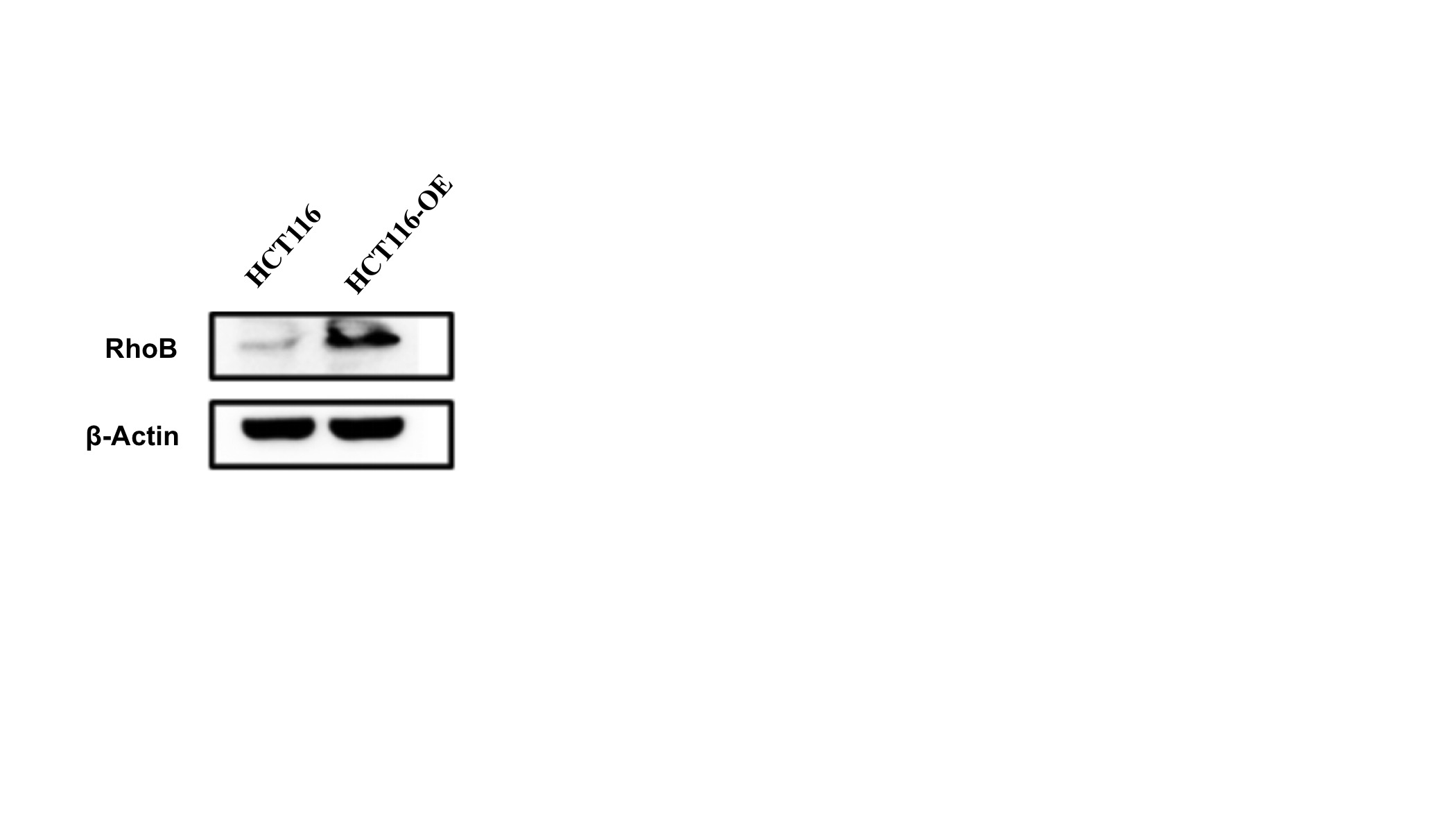


**Additional Figure 1.** Figure showing the protein level of RhoB in all the selected cell lines.

**Additional Figure 2.** Figure showing the statistical differences of RhoB WT *vs* KO/OE cell lines.


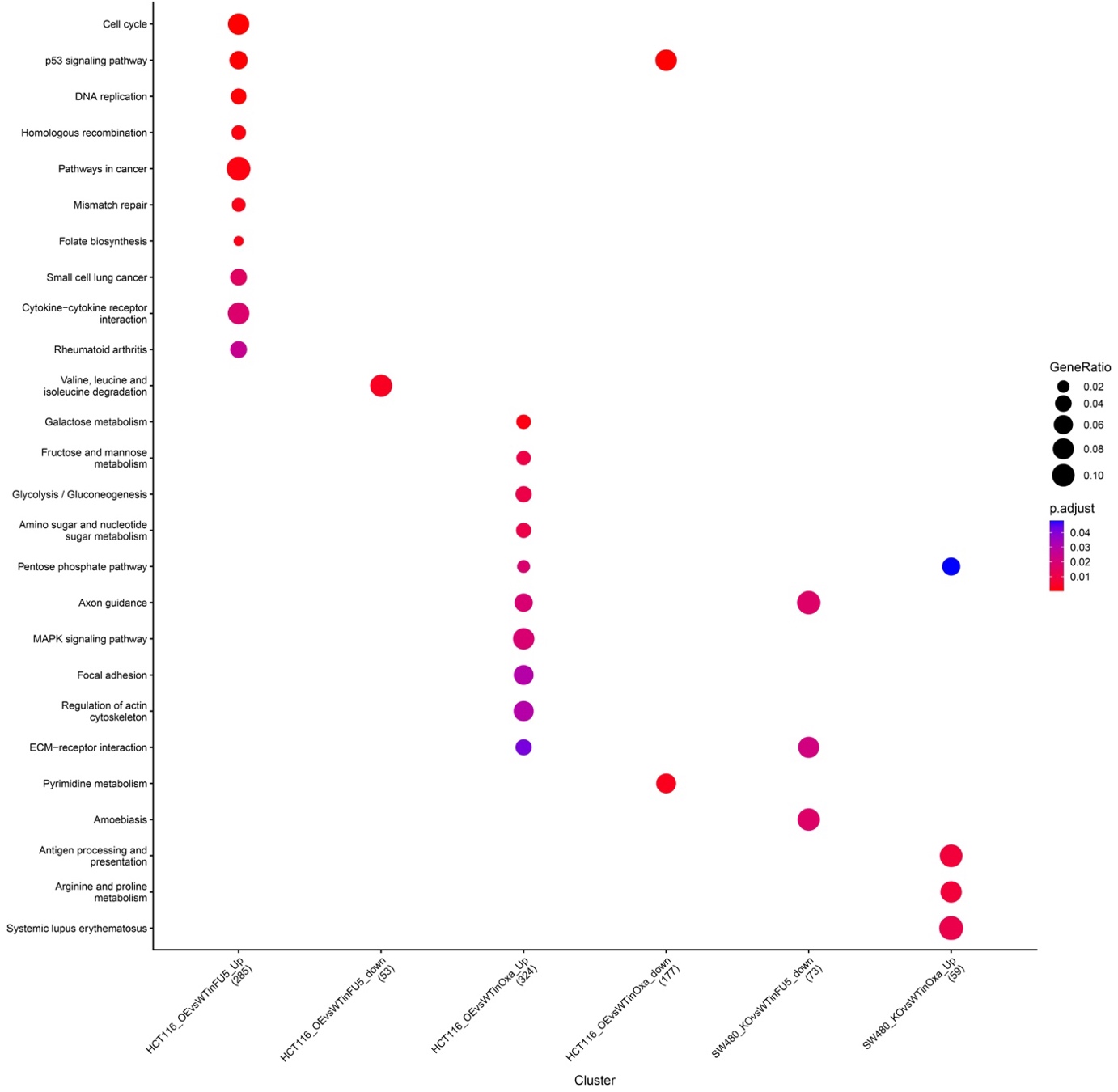


**Additional Figure 3.** KEGG pathway analysis of DEGs in HCT116 and SW480 cells after treatment with 5-fluorouracil (5-FU) and oxaliplatin (OXL).


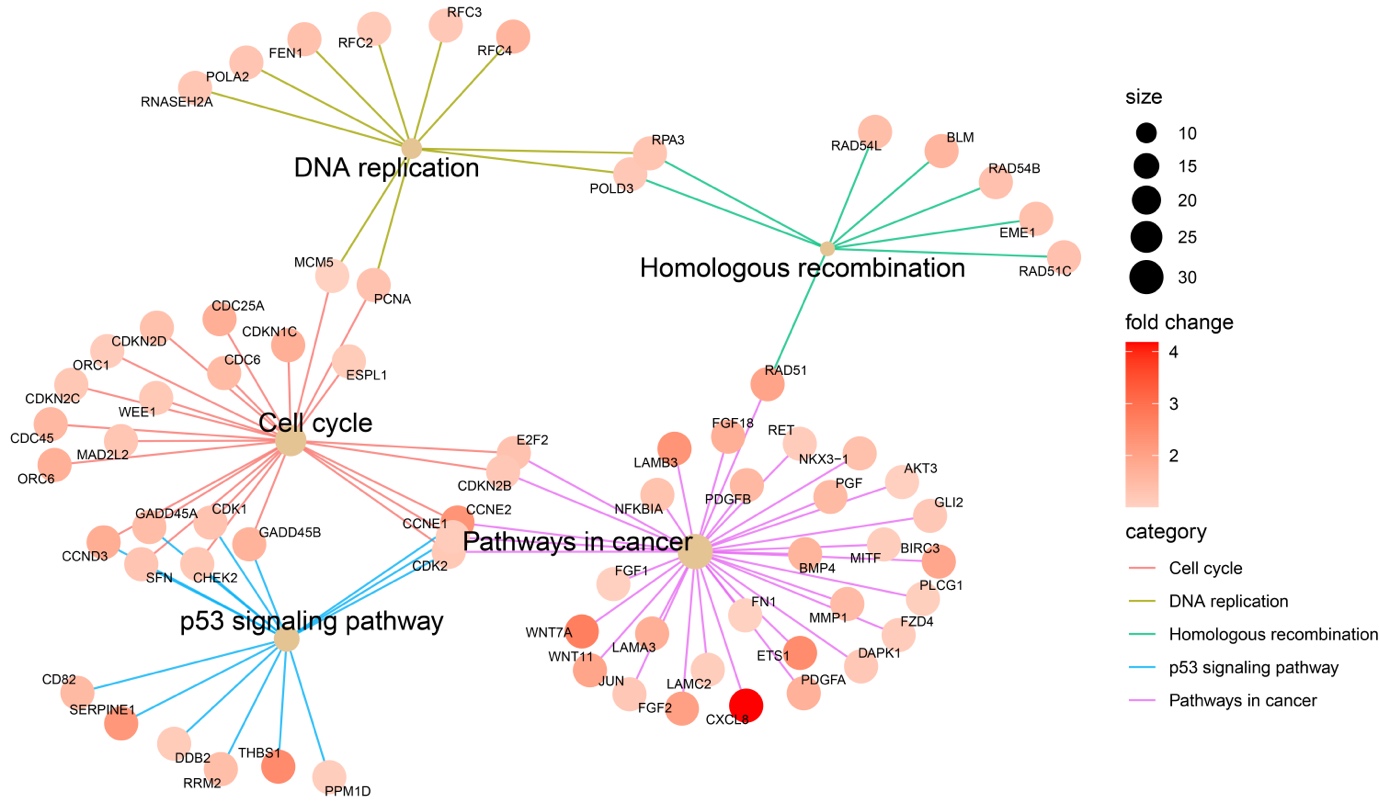


**Additional Figure 4.** KEGG pathway network analysis of DEGs in HCT116 cells after treatment with 5-fluorouracil (5-FU).

**
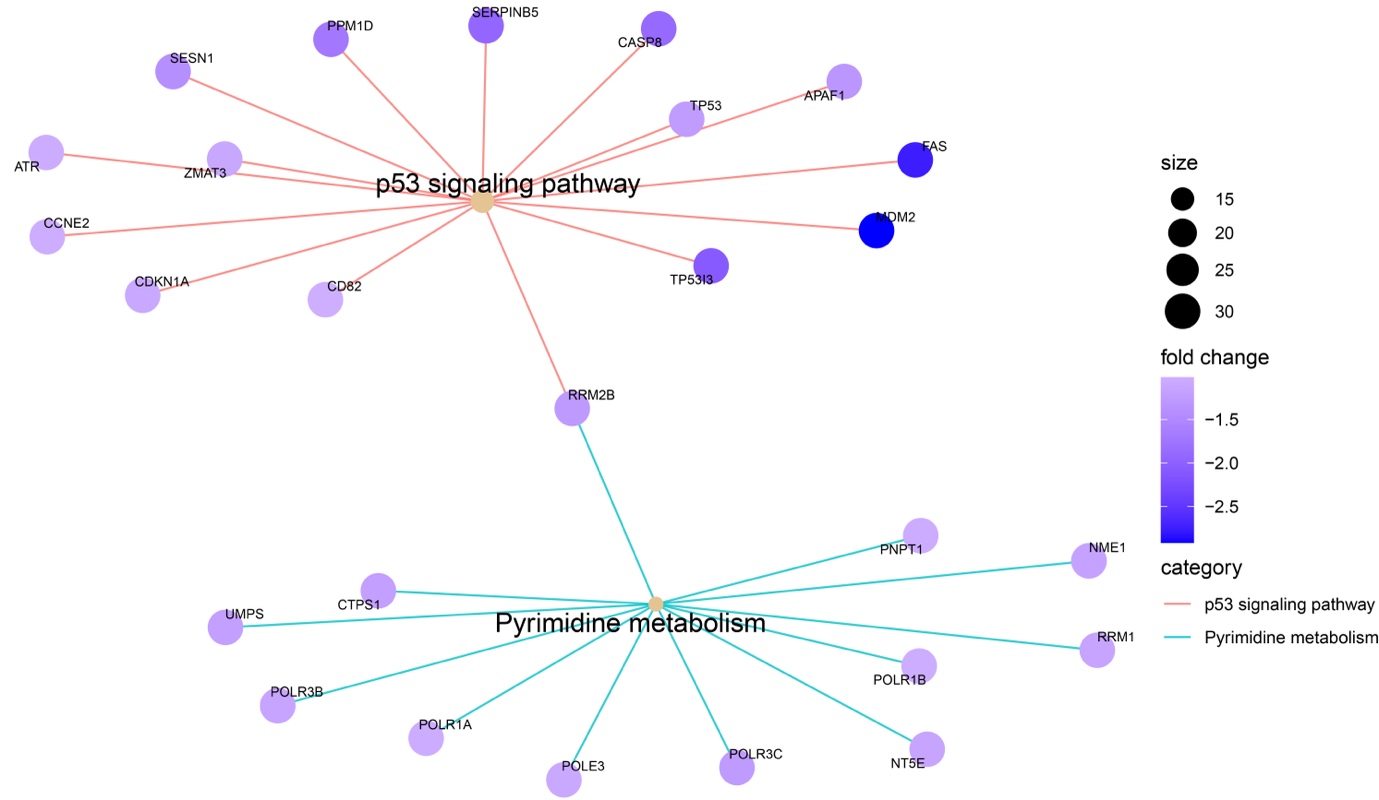
**

**Additional Figure 5.** KEGG pathway network analysis of DEGs in HCT116 cells after treatment with oxaliplatin (OXL).


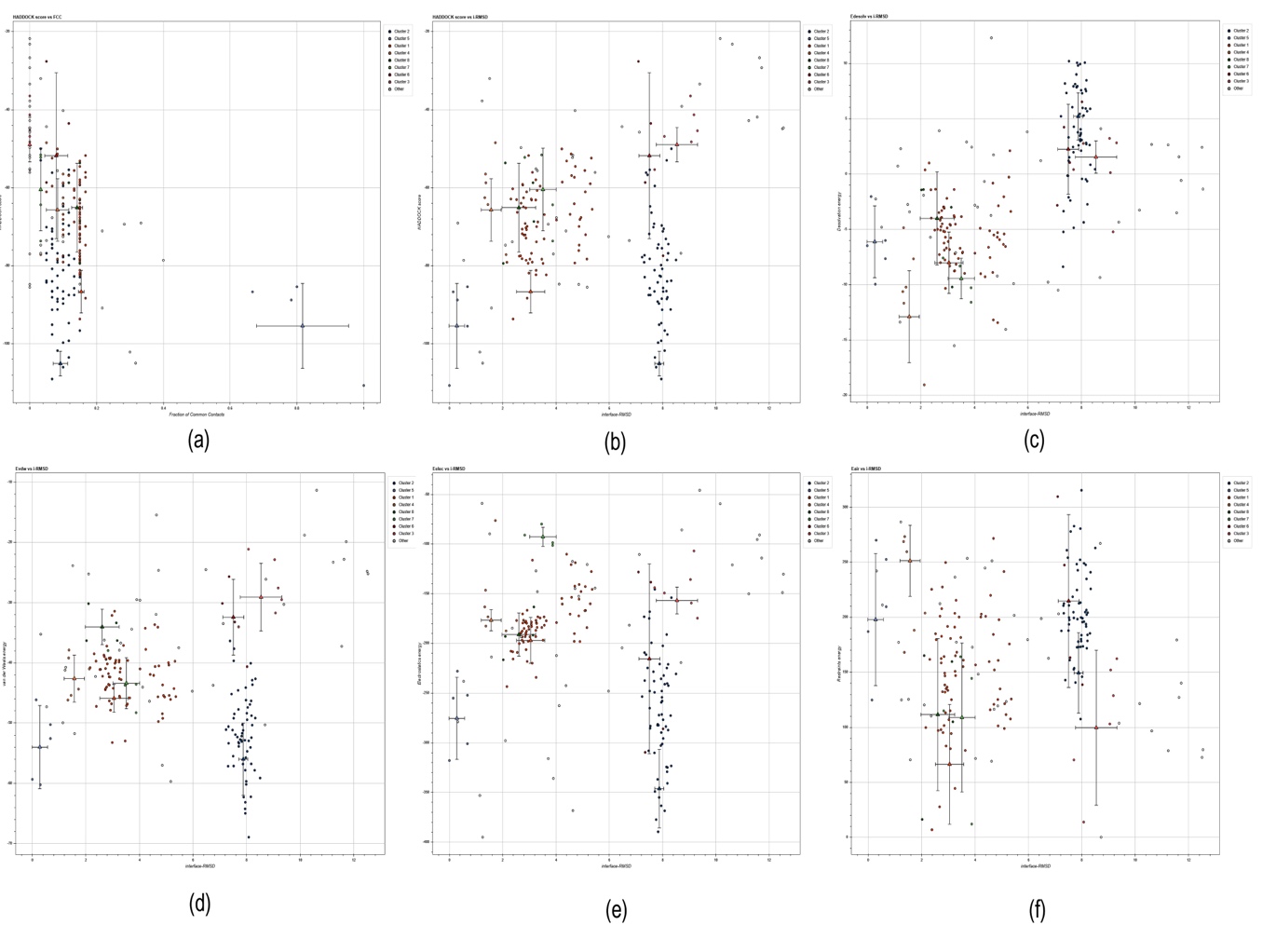


**Additional Figure 6.** The overall haddock score of all clusters generated for the RhoB and caspase 3 interaction.


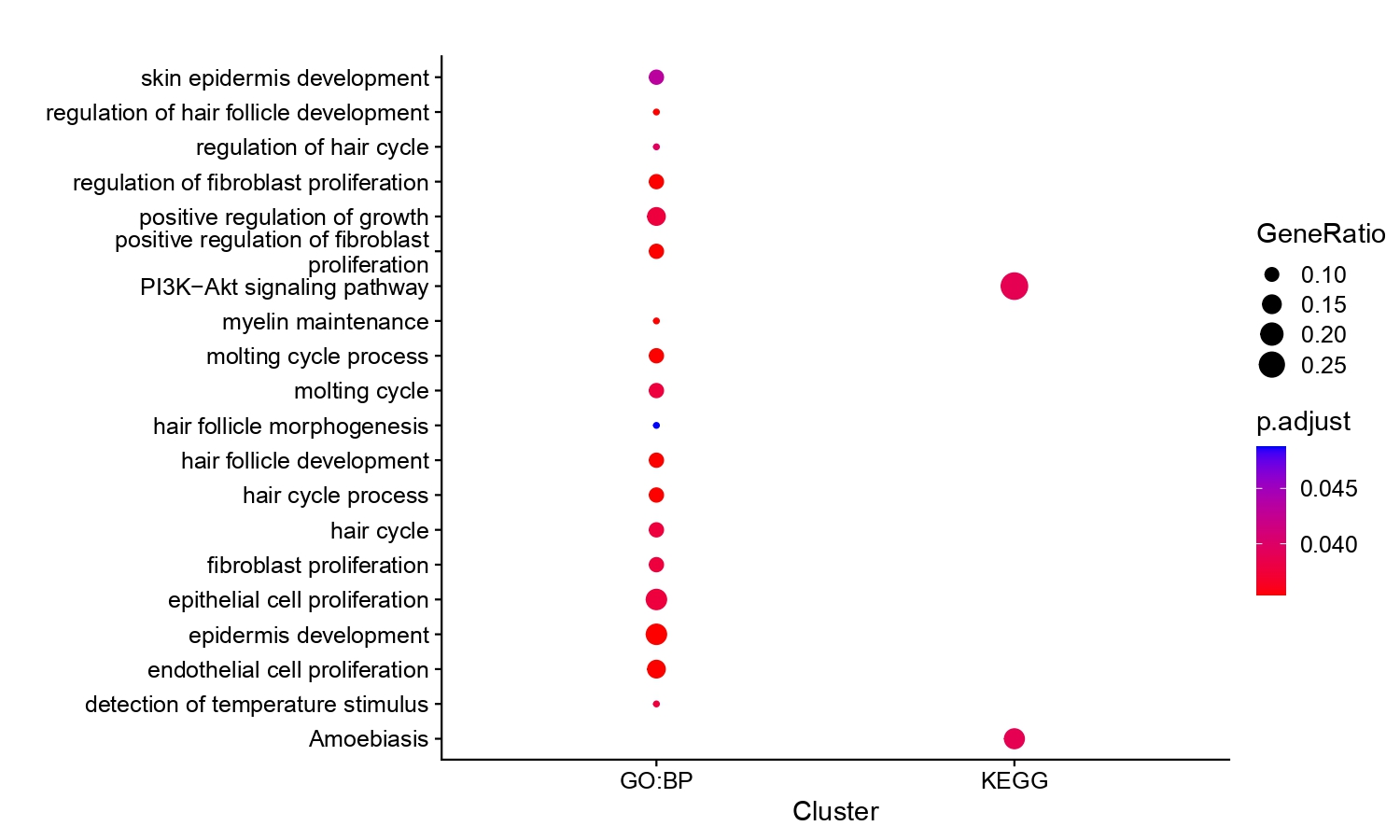


**Additional Figure 7.** Gene ontology **(**GO) biological process and KEGG pathway analysis of overlapping RhoB OE up-regulated and KO down-regulated genes.
