# Supplementary material for: RhoB expression associated with chemotherapy response and prognosis in colorectal cancer
Source: Cancer Cell Int. 2024 Feb 15;24:75. doi: 10.1186/s12935-024-03236-1 (PMC10867990; doi:10.1186/s12935-024-03236-1)
Supplement: Supplementary file 2 — Additional file 2: Table S1. Genome mapping summary of samples. Table S2. Characteristics of colorectal cancer patients. Table S4. Molecular docking analysis of RhoB and chemotherapy drugs with the interacting amino acid residues. Table S5. Molecular docking analysis between RhoB and caspase 3 proteins. Table S6. Protein–protein docking results of complete clusters generated from HADDOCK. Table S7. Interacting amino acid residues of RhoB and oxaliplatin. [file 12935_2024_3236_MOESM2_ESM.docx]

**Additional Table 1.** Genome mapping summary of samples

| Samples | Cell line | Treatment | Total | Total | Uniquely |
| --- | --- | --- | --- | --- | --- |
|  |  |  | Clean Reads | Mapping Ratio | Mapping Ratio |
| HCT116-OE_D1_controlA | HCT116-RhoB overexpression | control | 27853958 | 90.53% | 72.32% |
| HCT116-OE_D2_controlA | HCT116-RhoB overexpression | control | 27853646 | 90.64% | 72.07% |
| HCT116-OE_D3_5-fuA | HCT116-RhoB overexpression | 5-FU | 27865516 | 90.65% | 73.63% |
| HCT116-OE_D4_5-fuA | HCT116-RhoB overexpression | 5-Fu | 27982492 | 90.79% | 73.03% |
| HCT116-OE_D5_oxlA | HCT116-RhoB overexpression | oxaliplatin | 27970070 | 91.24% | 71.91% |
| HCT116-OE_D6_oxlA | HCT116-RhoB overexpression | oxaliplatin | 28011515 | 90.48% | 71.54% |
| HCT116_C1_controlA | HCT116-WT | control | 27867025 | 94.66% | 75.04% |
| HCT116_C2_controlA | HCT116-WT | control | 27884883 | 94.68% | 75.20% |
| HCT116_C3_5-fuA | HCT116-WT | 5-FU | 27782527 | 94.04% | 74.34% |
| HCT116_C4_5-fuA | HCT116-WT | 5-FU | 27867194 | 93.84% | 74.51% |
| HCT116_C5_oxlA | HCT116-WT | oxaliplatin | 27780286 | 94.15% | 74.96% |
| HCT116_C6_oxlA | HCT116-WT | oxaliplatin | 27814640 | 93.63% | 75.27% |
| SW480-KO_B1_controlA | SW480-RhoB knockout | Control | 26765635 | 94.44% | 77.02% |
| SW480-KO_B2_controlA | SW480-RhoB knockout | Control | 27901145 | 94.33% | 76.34% |
| SW480-KO_B3_5-fuA | SW480-RhoB Knockout | 5-FU | 27923968 | 93.62% | 76.24% |
| SW480-KO_B4_5-fuA | SW480-RhoB Knockout | 5-FU | 28011409 | 94.44% | 76.57% |
| SW480-KO_B5_oxlA | SW480-RhoB Knockout | Oxaliplatin | 27832013 | 94.46% | 75.73% |
| SW480-KO_B6_oxlA | SW480-RhoB Knockout | oxaliplatin | 27882777 | 94.24% | 75.65% |
| SW480_A1_controlA | SW480-WT | Control | 23672009 | 94.60% | 77.51% |
| SW480_A2_controlA | SW480-WT | control | 22829952 | 94.87% | 78.04% |
| SW480_A3_5-fuA | SW480-WT | 5-FU | 28265865 | 94.35% | 77.64% |
| SW480_A4_5-fuA | SW480-WT | 5-FU | 24975741 | 94.90% | 78.71% |
| SW480_A5_oxlA | SW480-WT | oxaliplatin | 23325619 | 94.73% | 78.30% |
| SW480_A6_oxlA | SW480-WT | oxaliplatin | 21839943 | 94.21% | 77.59% |

**Additional Table 2.** Characteristics of colorectal cancer patients

| **Characteristics** | **Number** | **Percentage** |
| --- | --- | --- |
| **Gender** |  |  |
| Male | 131 | 50% |
| Female | 129 | 50% |
| **Age at diagnosis (years)** |  |  |
| ≤ 70 | 126 | 48% |
| > 70 | 134 | 52% |
| **Tumor location** |  |  |
| Colon | 153 | 59% |
| Rectum | 106 | 41% |
| Rectosigmoid junction | 1 | <1% |
| **TNM stage** |  |  |
| I | 36 | 14% |
| II | 85 | 33% |
| III | 107 | 41% |
| IV | 32 | 12% |
| **Differentiation grade** |  |  |
| Good | 51 | 20% |
| Moderate | 141 | 54% |
| Poor | 68 | 26% |

**Additional Table 4.** Molecular docking analysis of RhoB and chemotherapy drugs with the interacting amino acid residues

| Protein name | Ligand | No. of bonding/interaction | Interacting amino acid | Binding affinity  (kcal/mol) |
| --- | --- | --- | --- | --- |
| RhoB | 5-FU | 3 hydrogen bond | ASP 120 | -5.2 |
|  |  |  | ALA 161 |  |
|  |  |  | LYS 162 |  |
|  |  | 2 Hydrophobic interaction | PHE 30 |  |
|  |  |  | LYS 118 |  |
|  | Oxaliplatin | 2 Hydrogen bond | GLY 17 | -7.8 |
|  |  |  | CYS 159 |  |
|  |  | 6 Van der Waals  interaction | LEU 21 |  |
|  |  |  | CYS 20 |  |
|  |  |  | PHE 30 |  |
|  |  |  | ASN 117 |  |
|  |  |  | ASP 120 |  |
|  |  |  | LYS 161 |  |

**Additional Table 5.** Molecular docking analysis between RhoB and caspase 3 proteins

| Term | Value |  |
| --- | --- | --- |
| HADDOCK score | -105.1 ± 2.7 |  |
| Cluster size | 60 |  |
| RMSD from the overall lowest-energy structure | 12.4 ± 0.3 |  |
| Van der Waals energy | -56.0 ± 5.2 |  |
| Electrostatic energy | -346.2 ± 34.2 |  |
| Desolvation energy | 5.2 ± 1.8 |  |
| Restraints violation energy | 149.4 ± 31.7 |  |
| Buried Surface Area | 2217.2 ± 130.5 |  |
| Z-Score | -1.7 |  |
| Term | Value | |
| HADDOCK score | -105.1 ± 2.7 | |
| Cluster size | 60 | |
| RMSD from the overall lowest-energy structure | 12.4 ± 0.3 | |
| Van der Waals energy | -56.0 ± 5.2 | |
| Electrostatic energy | -346.2 ± 34.2 | |
| Desolvation energy | 5.2 ± 1.8 | |
| Restraints violation energy | 149.4 ± 31.7 | |
| Buried Surface Area | 2217.2 ± 130.5 | |
| Z-Score | -1.7 | |

**Additional Table 6.** Protein-protein docking results of complete clusters generated from HADDOCK

**Cluster 1**

| Term | Value |
| --- | --- |
| HADDOCK score | -86.7 ± 4.7 |
| Cluster size | 73 |
| RMSD from the overall lowest-energy structure | 17.0 ± 0.4 |
| Van der Waals energy | -45.9 ± 2.0 |
| Electrostatic energy | -197.0 ± 20.0 |
| Desolvation energy | -8.0 ± 2.4 |
| Restraints violation energy | 66.4 ± 47.1 |
| Buried Surface Area | 1361.6 ± 48.9 |
| Z-Score | -0.7 |

**Cluster 2**

| Term | Value |
| --- | --- |
| HADDOCK score | -105.1 ± 2.7 |
| Cluster size | 60 |
| RMSD from the overall lowest-energy structure | 12.4 ± 0.3 |
| Van der Waals energy | -56.0 ± 5.2 |
| Electrostatic energy | -346.2 ± 34.2 |
| Desolvation energy | 5.2 ± 1.8 |
| Restraints violation energy | 149.4 ± 31.7 |
| Buried Surface Area | 2217.2 ± 130.5 |
| Z-Score | -1.7 |
| Term | Value |
| HADDOCK score | -105.1 ± 2.7 |
| Cluster size | 60 |
| RMSD from the overall lowest-energy structure | 12.4 ± 0.3 |
| Van der Waals energy | -56.0 ± 5.2 |
| Electrostatic energy | -346.2 ± 34.2 |
| Desolvation energy | 5.2 ± 1.8 |
| Restraints violation energy | 149.4 ± 31.7 |
| Buried Surface Area | 2217.2 ± 130.5 |
| Z-Score | -1.7 |

**Cluster 3**

| Term | Value |
| --- | --- |
| sHADDOCK score | -49.0 ± 3.8 |
| Cluster size | 6 |
| RMSD from the overall lowest-energy structure | 19.7 ± 0.3 |
| Van der Waals energy | -29.1 ± 4.9 |
| Electrostatic energy | -156.8 ± 11.7 |
| Desolvation energy | 1.5 ± 1.3 |
| Restraints violation energy | 99.7 ± 61.0 |
| Buried Surface Area | 1298.3 ± 85.3 |
| Z-Score | 1.2 |

**Cluster 4**

| Term | Value |
| --- | --- |
| HADDOCK score | -65.7 ± 6.9 |
| Cluster size | 6 |
| RMSD from the overall lowest-energy structure | 16.3 ± 0.5 |
| Van der Waals energy | -42.6 ± 3.4 |
| Electrostatic energy | -176.6 ± 9.2 |
| Desolvation energy | -12.9 ± 3.6 |
| Restraints violation energy | 251.3 ± 27.9 |
| Buried Surface Area | 1637.5 ± 143.6 |
| Z-Score | 0.3 |

**Cluster 5**

| Term | Value |
| --- | --- |
| HADDOCK score | -95.4 ± 9.4 |
| Cluster size | 5 |
| RMSD from the overall lowest-energy structure | 0.8 ± 0.4 |
| Van der Waals energy | -54.0 ± 6.0 |
| Electrostatic energy | -275.5 ± 35.8 |
| Desolvation energy | -6.1 ± 2.8 |
| Restraints violation energy | 197.8 ± 51.9 |
| Buried Surface Area | 1899.1 ± 100.3 |
| Z-Score | -1.2 |

**Cluster 6**

| Term | Value |
| --- | --- |
| HADDOCK score | -51.8 ± 18.4 |
| Cluster size | 4 |
| RMSD from the overall lowest-energy structure | 13.3 ± 0.4 |
| Van der Waals energy | -32.4 ± 5.5 |
| Electrostatic energy | -215.5 ± 82.8 |
| Desolvation energy | 2.2 ± 3.5 |
| Restraints violation energy | 214.7 ± 68.0 |
| Buried Surface Area | 1628.0 ± 150.1 |
| Z-Score | 1.1 |

**Cluster 7**

| Term | Value |
| --- | --- |
| HADDOCK score | -60.5 ± 9.2 |
| Cluster size | 4 |
| RMSD from the overall lowest-energy structure | 18.5 ± 0.2 |
| Van der Waals energy | -43.4 ± 3.7 |
| Electrostatic energy | -92.7 ± 8.3 |
| Desolvation energy | -9.4 ± 1.6 |
| Restraints violation energy | 108.9 ± 58.6 |
| Buried Surface Area | 1442.8 ± 73.3 |
| Z-Score | 0.6 |

**Cluster 8**

| Term | Value |
| --- | --- |
| HADDOCK score | -65.1 ± 9.9 |
| Cluster size | 4 |
| RMSD from the overall lowest-energy structure | 16.8 ± 0.4 |
| Van der Waals energy | -34.0 ± 2.5 |
| Electrostatic energy | -191.1 ± 18.9 |
| Desolvation energy | -4.0 ± 3.6 |
| Restraints violation energy | 111.5 ± 59.8 |
| Buried Surface Area | 1238.0 ± 52.3 |
| Z-Score | 0.4 |

**Additional Table 7.** Interacting amino acid residues of RhoB and oxaliplatin

|  |  |  |  |  |  |
| --- | --- | --- | --- | --- | --- |
| PHE | 39 | A | PRO | 133 | B |
| GLY | 14 | A | LEU | 168 | B |
| LEU | 69 | A | SER | 63 | B |
| GLU | 40 | A | MET | 61 | B |
| GLU | 40 | A | GLU | 123 | B |
| PHE | 39 | A | ILE | 126 | B |
| LYS | 27 | A | ILE | 126 | B |
| GLN | 63 | A | THR | 62 | B |
| ASP | 87 | A | GLU | 167 | B |
| ASN | 94 | A | THR | 255 | B |
| ASP | 13 | A | THR | 255 | B |
| SER | 73 | A | GLY | 60 | B |
| ALA | 61 | A | GLY | 60 | B |
| GLN | 63 | A | GLY | 60 | B |
| GLY | 14 | A | THR | 166 | B |
| CYS | 16 | A | THR | 166 | B |
| ASP | 65 | A | PHE | 256 | B |
| GLY | 14 | A | THR | 255 | B |
| ASP | 13 | A | PHE | 256 | B |
| TYR | 66 | A | SER | 65 | B |
| LEU | 69 | A | THR | 62 | B |
| PRO | 31 | A | GLU | 124 | B |
| GLU | 40 | A | PHE | 128 | B |
| ALA | 61 | A | TYR | 204 | B |
| CYS | 16 | A | GLU | 167 | B |
| ASN | 41 | A | THR | 59 | B |
| ASN | 94 | A | ALA | 254 | B |
| GLU | 64 | A | PHE | 256 | B |
| TYR | 66 | A | SER | 209 | B |
| ASN | 41 | A | GLY | 60 | B |
| ASP | 59 | A | GLY | 60 | B |
| TRP | 99 | A | ASP | 253 | B |
| TYR | 66 | A | ARG | 207 | B |
| ALA | 61 | A | THR | 62 | B |
| THR | 19 | A | GLU | 123 | B |
| ASN | 41 | A | SER | 58 | B |
| CYS | 20 | A | THR | 166 | B |
| SER | 91 | A | THR | 255 | B |
| GLY | 62 | A | PHE | 256 | B |
| PHE | 39 | A | MET | 61 | B |
| GLU | 130 | A | ILE | 172 | B |
| GLU | 40 | A | THR | 59 | B |
| GLU | 40 | A | ARG | 93 | B |
| PHE | 39 | A | GLU | 123 | B |
| TRP | 58 | A | GLY | 60 | B |
| ALA | 15 | A | GLU | 167 | B |
| CYS | 20 | A | GLY | 165 | B |
| LYS | 18 | A | TYR | 204 | B |
| ALA | 15 | A | TYR | 204 | B |
| LYS | 118 | A | THR | 166 | B |
| GLY | 14 | A | PHE | 256 | B |
| ASP | 65 | A | PHE | 252 | B |
| GLU | 130 | A | GLY | 171 | B |
| LEU | 72 | A | LYS | 57 | B |
| ASP | 65 | A | ASP | 253 | B |
| GLU | 40 | A | PRO | 133 | B |
| TYR | 66 | A | SER | 63 | B |
| ASP | 13 | A | ASP | 253 | B |
| GLU | 40 | A | SER | 58 | B |
| THR | 19 | A | TYR | 204 | B |
| LYS | 118 | A | GLU | 167 | B |
| PHE | 39 | A | PHE | 128 | B |
| ASP | 90 | A | THR | 255 | B |
| ALA | 15 | A | LEU | 168 | B |
| GLY | 62 | A | THR | 62 | B |
| GLY | 17 | A | THR | 166 | B |
| SER | 73 | A | LYS | 57 | B |
| GLU | 64 | A | ASP | 253 | B |
| ARG | 133 | A | ILE | 172 | B |
| GLU | 40 | A | GLY | 132 | B |
| TRP | 58 | A | LYS | 57 | B |
| LYS | 18 | A | THR | 166 | B |
| TYR | 66 | A | THR | 62 | B |
| ALA | 15 | A | THR | 166 | B |
| ALA | 61 | A | THR | 166 | B |
| ALA | 15 | A | THR | 255 | B |
| LYS | 98 | A | ASP | 253 | B |
| ASP | 90 | A | ALA | 254 | B |
| LYS | 27 | A | GLU | 123 | B |
| LEU | 121 | A | GLU | 167 | B |
| GLY | 62 | A | TYR | 204 | B |
| THR | 19 | A | THR | 166 | B |
| ASP | 59 | A | MET | 61 | B |
| ASN | 41 | A | LYS | 57 | B |
| PHE | 39 | A | THR | 59 | B |
| SER | 88 | A | THR | 255 | B |
| ASP | 13 | A | TYR | 204 | B |
| GLY | 14 | A | TYR | 204 | B |
| THR | 19 | A | GLY | 165 | B |
| GLU | 40 | A | ASN | 131 | B |
| GLY | 14 | A | ASP | 253 | B |
| GLU | 64 | A | TYR | 204 | B |
| ALA | 61 | A | MET | 61 | B |
| GLN | 63 | A | MET | 61 | B |
| ILE | 23 | A | GLU | 123 | B |
| ASP | 65 | A | SER | 251 | B |
